# Assessing Species Boundaries Using Multilocus Species Delimitation in a Morphologically Conserved Group of Neotropical Freshwater Fishes, the *Poecilia sphenops* Species Complex (Poeciliidae)

**DOI:** 10.1371/journal.pone.0121139

**Published:** 2015-04-07

**Authors:** Justin C. Bagley, Fernando Alda, M. Florencia Breitman, Eldredge Bermingham, Eric P. van den Berghe, Jerald B. Johnson

**Affiliations:** 1 Evolutionary Ecology Laboratories, Department of Biology, Brigham Young University, Provo, Utah, 84602, United States of America; 2 Smithsonian Tropical Research Institute, Balboa, Panamá; 3 Centro Nacional Patagónico (CENPAT-CONICET), U9120ACD, Puerto Madryn, Chubut, Argentina; 4 Centro Zamorano de Biodiversidad, Departamento de Ambiente y Desarrollo, Zamorano University, Tegucigalpa, Honduras; 5 Monte L. Bean Life Science Museum, Brigham Young University, Provo, Utah, 84602, United States of America; University of British Columbia Okanagan, CANADA

## Abstract

Accurately delimiting species is fundamentally important for understanding species diversity and distributions and devising effective strategies to conserve biodiversity. However, species delimitation is problematic in many taxa, including ‘non-adaptive radiations’ containing morphologically cryptic lineages. Fortunately, coalescent-based species delimitation methods hold promise for objectively estimating species limits in such radiations, using multilocus genetic data. Using coalescent-based approaches, we delimit species and infer evolutionary relationships in a morphologically conserved group of Central American freshwater fishes, the *Poecilia sphenops* species complex. Phylogenetic analyses of multiple genetic markers (sequences of two mitochondrial DNA genes and five nuclear loci) from 10/15 species and genetic lineages recognized in the group support the *P*. *sphenops* species complex as monophyletic with respect to outgroups, with eight mitochondrial ‘major-lineages’ diverged by ≥2% pairwise genetic distances. From general mixed Yule-coalescent models, we discovered (conservatively) 10 species within our concatenated mitochondrial DNA dataset, 9 of which were strongly supported by subsequent multilocus Bayesian species delimitation and species tree analyses. Results suggested species-level diversity is underestimated or overestimated by at least ~15% in different lineages in the complex. Nonparametric statistics and coalescent simulations indicate genealogical discordance among our gene tree results has mainly derived from interspecific hybridization in the nuclear genome. However, mitochondrial DNA show little evidence for introgression, and our species delimitation results appear robust to effects of this process. Overall, our findings support the utility of combining multiple lines of genetic evidence and broad phylogeographical sampling to discover and validate species using coalescent-based methods. Our study also highlights the importance of testing for hybridization *versus* incomplete lineage sorting, which aids inference of not only species limits but also evolutionary processes influencing genetic diversity.

## Introduction

Species are widely used as fundamental units of analysis in biogeography, ecology, and evolutionary biology [1–3]. Species also figure prominently in biodiversity assessments and conservation recovery programs [4]. Therefore, species delimitation, the practice of determining species boundaries and discovering new species, is of fundamental importance for understanding species diversity and distributions, and devising effective strategies to conserve biodiversity [5–7]. By contrast, inaccurately classifying individuals or populations to species could result in erroneous inferences in any analysis requiring *a priori* designation of species limits, such as comparative analyses of diversification [8,9], or misallocation of conservation resources and loss of species (e.g. under the U.S. Endangered Species Act of 1973; [5]).

Although species are universally recognized as metapopulation lineages distinct from other such aggregates (‘general lineage concept’, or GLC; [10–12]), determining which operational criteria should be used to assign individuals to species is a major problem in species delimitation. Independently applying operational criteria with different philosophical bases often yields incongruent species boundaries [5,13,14]. In turn, inconsistent application of operational species concepts creates unstable taxonomy, injecting taxonomic uncertainty into efforts at species enumeration e.g. [6]. In light of practical difficulties presented by applying alternative operational criteria, there is a growing consensus that multiple perspectives from different data-types or analyses are necessary to accurately delimit species, through ‘integrative taxonomy’, e.g. uniting classical morphology, phylogenetics, and ecological data and modeling [15–17].

The present surge of interest in integrative taxonomy has shifted biologists’ focus away from using single operational criteria to sampling multiple lines of evidence, which ideally yields more robust species delimitations [5,17]. However, integrating morphology with genetic data is notoriously difficult in a variety of contexts. Some examples include: (1) morphologically conserved, ‘non-adaptive radiations’ containing cryptic species [18–21]; (2) systems with high taxonomic uncertainty; (3) rapid and recent adaptive radiations [22,23]; and (4) taxa with porous species boundaries [24]. In the first two cases, morphological methods often fail to detect cryptic species and are prone to underestimate species diversity [8]; thus, integrative taxonomic approaches combining morphology with other data will likely yield discordant inferences promoting subjective interpretations. Reliance on morphology can also produce spurious phylogenetic inferences due to disruptive natural selection or insufficient character variation [5,25]. In the latter two cases, speciation can be incomplete or in its early stages, yielding limited genetic variation and higher likelihood of gene tree discordance due to introgressive hybridization e.g. [26] or incomplete lineage sorting (ILS; e.g. [27]). Also in such cases, ‘DNA barcoding’ and single-locus gene trees may fail to establish clear phylogenetic support for fixed differences in morphology among distinct lineages e.g. [22].

Recently, the growth of methods for analyzing DNA sequence data in a coalescent-based framework capable of accounting for confounding processes such as ILS [28] has sparked a ‘Renaissance’ in empirical species delimitation (reviewed by [5,29]). Various coalescent-based methods are now available that address different goals in species delimitation, including *de novo* species discovery [30–34], species validation [25,35,36], and assignment of unknown individuals to species e.g. [37]. However, these methods are united in using algorithms modeling evolutionary processes, including likelihood and Bayesian analyses, to identify independent evolutionary lineages as distinct species based on multilocus data and species trees or ‘guide trees’ [29,34]. Indeed, the rapid growth of these methods owes partly to the incorporation of new methods for species tree inference using the multispecies coalescent e.g. [38,39], which has also revolutionized phylogenetics [40]. Overall, the new wave of coalescent-based species delimitation methods greatly improves the rigor and objectivity of species delimitation, and holds promise for meeting the need for rapid biodiversity assessment and species descriptions [41–43] in light of the current global biodiversity crisis [44].

Although the field of coalescent-based species delimitation is in its infancy, its tools provide solutions to the problems of delimiting species in radiations at the extremes of morphological or genetic divergence (*sensu* [21], their Fig 1; at least cases 2 and 4 above). For example, aside from delimiting species in “easy-delimitation” scenarios (e.g. deeply diverged lineages with small population sizes; [45]), coalescent-based methods have proven useful for resolving species limits in studies of more difficult cases of morphologically cryptic radiations including trapdoor spiders [46], cave fishes [20], kingsnakes [47], sun skinks [21] and water monitors [48]. In particular, the ‘chimeric approach’ of developing preliminary species hypotheses using parametric or heuristic methods often applied to mitochondrial DNA (mtDNA), then validating these using Bayesian species delimitation with multiple genetic loci [25], appears to be a fruitful way forward (pioneered by Leaché & Fujita [43]; also see [20,21,49]). Under this approach, working hypotheses of species distributions are established and tested using multilocus data and methods taking ILS into account, and the results provide bases for subsequent tests of species morphological and ecological distinctiveness in an integrative taxonomy framework [5].

**Fig 1:**
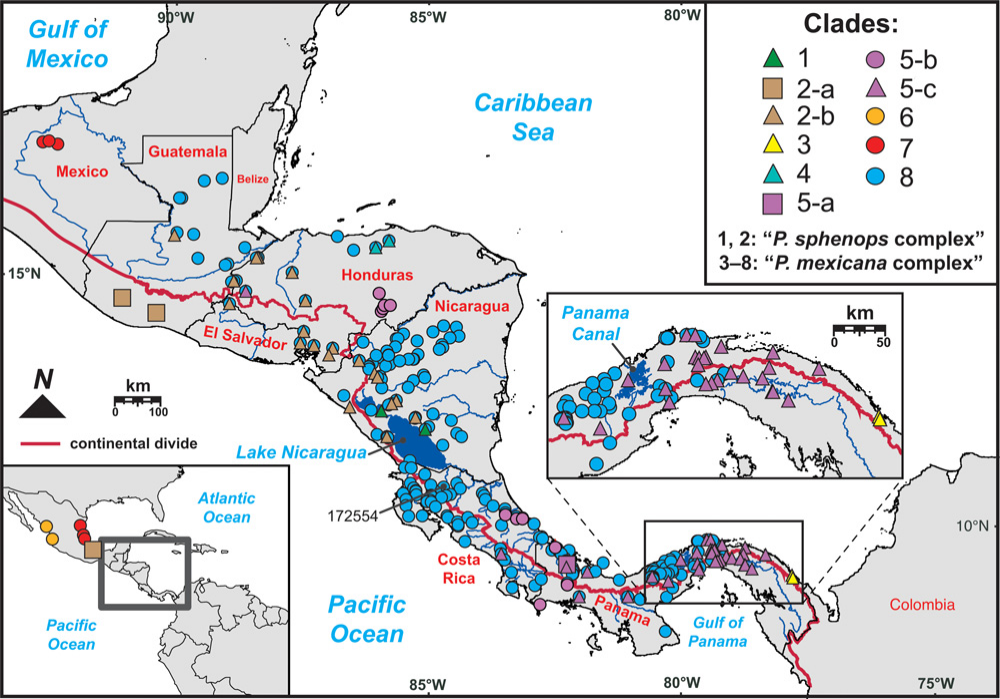
*Poecilia sphenops* species complex sampling localities and phylogeographical structuring throughout Central America. Sampling localities (dots) correspond to collections data in S1 Data and are colored according to phylogenetic clades or ‘major-lineages’ in Fig 2 and the upper right legend. Some localities for clades 2-a and 6 are shown in the overview map (bottom left). The legend also lists clades corresponding to two monophyletic sub-complexes within the complex *sensu lato*, supported here (see Results) and in previous studies [55,64]. The locality for one sample whose phylogenetic position fluctuated during analyses (172554) is indicated on the map. Regional context is given by geopolitical boundaries (country names in red) and the continental divide (red line).

In this study, we use a coalescent-based chimeric approach to delimit species and expand on previous knowledge of the patterns and processes of diversification in a morphologically conserved radiation—livebearing freshwater fishes in the *Poecilia sphenops* species complex (family Poeciliidae) [50,51]. Despite being among the most common members of regional fish communities in the Mesoamerica biodiversity hotspot [52–54], species limits and taxonomy are incompletely resolved in the group (reviewed by [55]). Here, we develop the most comprehensive sampling and multilocus sequencing from across the geographical distribution of the *P*. *sphenops* species complex to date, to delimit species and evaluate their evolutionary genetic relationships. Our objectives were (1) to develop preliminary species delimitation hypotheses using mtDNA; (2) to infer the species tree and timing of lineage diversification using relaxed molecular clocks; (3) to test species validity using multilocus Bayesian species delimitation; and (4) to test model fit and potential sources of gene tree discordance. We use our results to evaluate the validity of nominal taxa and cryptic genetic lineages currently recognized in this group, and to clarify species present distributions.

## Materials and Methods

### Systematic Background

The systematics of the genus *Poecilia* Bloch & Schneider 1801 has experienced multiple changes since its initial description, including redescriptions and synonymizations. The currently accepted taxonomy of *Poecilia* recognizes four subgenera: *Limia*, *Pamphorichthys*, *Lebistes*, and *Mollienesia* (*sensu* [56]). *Mollienesia* contains 15 to 25 species distributed from North to South America that fall into two species groups distinguished by differences in dorsal fin size and behavior—‘sail-fin’ and ‘short-fin’ species [50,51,56–58]. However, much taxonomic confusion in *Mollienesia* owes to their conserved morphology, which obscures interspecific variation; for example, diagnostic characters may overlap, and species display plasticity such that intraspecific phenotypic variance can outpace divergence between species [59,60]. Indeed, the morphologically conserved nature of *Mollienesia* led early workers to conclude that all short-fins represented ‘races’ or local variants of a single polytypic taxon, *P*. *sphenops* Valenciennes 1864, ranging geographically from the Río Grande drainage in northeastern Mexico to coastal Venezuela [61–63]. However, another more widely accepted view is that the short-fin group is composed of species with partly overlapping geographical ranges that constitute the ‘*P*. *sphenops* species complex’ [51,55,64,65].

The *P*. *sphenops* species complex is a monophyletic group of 13 described species that is widely distributed along Atlantic and Pacific slopes throughout Mexico and the Central American Neotropics, from the Río Grande through Panama [51,55]. Some authors suggest that this species complex can be further sub-divided into two sub-complexes based on morphology (tricuspid and unicuspid inner jaw teeth, respectively [50,55]) and mitochondrial DNA [55]: a ‘*P*. *sphenops* complex’ including species from the Pacific slope of Mexico through Central America, and a ‘*P*. *mexicana* complex’ including species from Atlantic coastal Mexico to Nicaragua [64,65]. The *P*. *sphenops* complex includes four described species (*P*. *chica*, *P*. *marcellinoi*, *P*. *maylandi*, and *P*. *sphenops*) and one molecularly-identified ‘candidate species’ closely related to *P*. *sphenops* (“*sphenops*” sp. 1 from Honduras and Nicaragua, in Alda *et al*. [55]). The *P*. *mexicana* complex includes nine described species (*P*. *butleri*, *P*. *catemaconis*, *P*. *gillii*, *P*. *hondurensis*, *P*. *mexicana*, *P*. *orri*, *P*. *sulphuraria*, and *P*. *teresae*) and one candidate species closely related to *P*. *gillii* (“*gillii*” sp. 2 from Río Acla, Panama [55]). Excluding the candidate species, nearly all of these taxa are currently recognized as valid species, except for *P*. *mexicana*. Based on a geographical analysis of morphological variation (despite not covering the full geographical range of *P*. *mexicana*), two subspecies have been recognized within *P*. *mexicana*: *P*. *m*. *mexicana*, and *P*. *m*. *limantouri* restricted to the northeastern Gulf coastal drainages of Mexico [66]. Although these two subspecies have been formally evaluated and described, it seems to the best of our knowledge that this taxonomic change has been ignored by other workers in the literature (or not formally corrected if mistaken) until recently [67,68], where the subspecies of *P*. *mexicana* have been listed but not formally compared.

S1 Table summarizes the proposed taxonomic arrangements, tooth morphology, and currently recognized geographical distributions of species in the *P*. *sphenops* species complex. Although some species (e.g. *P*. *catemaconis* in Lake Catemaco, Mexico) are local endemics with restricted distributions, several others (e.g. *P*. *sphenops*) have relatively large ranges and occur along Atlantic and Pacific slopes (S1 Table). Indeed, the large distribution of some species hinders taxonomic identification because intraspecific morphological gradients or local differentiations are common, and this has been hypothesized to promote character displacement when taxa in the complex occur in sympatry with one another [69].

### Ethics Statement

Permission to undertake fieldwork for this study was obtained through permits issued to JCB and JBJ in Nicaragua by MARENA (Ministerio de Ambiente y Recursos Naturales; DGPN/DB-IC-009-2012; DGPN/DB-21-2012) and in Costa Rica by SINAC-MINAET (Ministerio de Ambiente Energía y Telecomunicaciones; Resolución No. 030-2010-SINAC, Resolución No. 134-2012-SINAC). New specimens were obtained through these collections under Brigham Young University Institutional Animal Care and Use Committee (IACUC) approval #12–0701. Other samples were obtained through government-authorized fieldwork conducted in previous studies ([55,70]; supplementary S1 Data).

### Taxon Sampling and Sequencing

We sampled populations of *Poecilia* through field expeditions conducted in Central America, and from the fish tissue archives of our laboratories, the STRI Neotropical Fish Collection (STRI-NFC) and the Monte L. Bean Life Science Museum Fish Collection (BYU). In total, we sampled 873 *Poecilia* individuals from 260 localities (Fig 1; S1 Data). We identified samples to species based on their different combinations of morphology and geographic distributions, following published taxonomy and biogeography studies [50,55,66]. Voucher specimens are deposited at STRI-NFC and BYU.

Of the 13 described species in the *P*. *sphenops* species complex, we sampled eight species (*P*. *butleri*, *P*. *catemaconis*, *P*. *gillii*, *P*. *hondurensis*, *P*. *mexicana*, *P*. *orri*, *P*. *salvatoris*, and *P*. *sphenops*) and two exclusive mtDNA lineages, or ‘operational taxonomic units’ (OTUs), identified from recent molecular phylogenetic analyses by Alda *et al*. [55] (“*sphenops*” sp. 1 and “*gillii*” sp. 2). We augmented our sampling with sequences of *P*. *sulphuraria*, *P*. *thermalis*, and the subspecies *P*. *mexicana limantouri* from previous studies (see below) and tested each of these taxa as species-level OTUs. Taking the general lineage concept of species and reconsidering morphology and genetics using a phylogenetic criterion for species identification [11,12], we considered “*P*. *orri*” samples forming an exclusive genetic lineage from Río Patuca, Honduras in [55] to be a novel OTU, or candidate species, that we refer to as *P*. sp. “Patuca”. One motivation for this was that *P*. sp. “Patuca” males possess hooks on their gonopodia (anal fins modified into intromittent organs), whereas a lack of such hooks is a diagnostic character for *P*. *orri* [71]. We tested this hypothesis by also including in our analyses samples confidently assigned to *P*. *orri* from Roatan, the next major island adjacent to (~10 km from) the original type locality of *P*. *orri* at Bonacca Island off the northern Honduras coast [71]. Instead of rigorously evaluating species boundaries using morphological data, we used species diagnoses based on current taxonomy and our interpretation of published phylogenetic relationships as our null hypotheses. This study design amounts to testing hypotheses of species limits based on morphological (e.g. [72]) and/or phylogenetic criteria (genealogical or diagnostic, as in [10,12]) for empirical recognition of species. Our final dataset encompassed 10 out of 15 putative species-level lineages or OTUs recognized in the group (S1 Table), plus two subspecies, and most of the geographic range of the complex. We also sampled four poeciliid outgroups: *P*. *latipinna*, *P*. *latipunctata* (Mexico), *Limia perugiae* (Hispaniola), and *P*. *caucana* (Panama) samples; yet we analyzed up to 15 outgroup taxa, including samples from genomic repositories, to obtain phylogenetic calibration points (S1 Appendix).

We extracted whole genomic DNA from tissue samples using Qiagen DNeasy Tissue Kits (QIAGEN Sciences, Maryland, USA) and sequenced the protein-coding mitochondrial cytochrome *b* (cyt*b*) gene for every individual, except problematic *P*. *orri* and *P*. *salvatoris* samples, using two primers flanking the gene (Table 1). To obtain additional mtDNA characters for analysis, we sequenced the mtDNA cytochrome oxidase 1 (*cox1*) gene for individuals chosen to maximize geographic and phylogenetic coverage of mtDNA major-lineages, using fish ‘barcode’ primers (Table 1). In pilot analyses, *cox1* subsampling improved the mtDNA gene tree topology by increasing nodal support (data not shown); however, it appeared that sequencing every individual for *cox1* would not provide any added benefit, as expected when subsampling linked mitochondrial genes [24,73]. We also sequenced five nuclear DNA (nDNA) loci: ribosomal protein S7 (*RPS7*; introns 1 and 2 and exon 2); muscle-type lactate dehydrogenase (*ldh-A*); tyrosine-kinase class oncogenes, X*-src* and X*-yes*; and glycosyltransferase (*Glyt*). Because they showed limited genetic variation and we could not sequence every individual for each locus, we sequenced the nuclear loci for subsamples chosen to maximize geographic and phylogenetic coverage (1–5 individuals from each mtDNA major-lineage), which we used for species tree and species validation analyses. With the exception of *ldh-A*, we amplified nuclear loci via nested polymerase chain reactions (PCR), as described in Table 1 and [74]. We purified PCR products using a Montage PCR 96 plate (Millipore, Billerica, MA, USA). Sequences were obtained via cycle sequencing with Big Dye 3.1 dye terminator chemistry using 1/16^th^ reaction size and the manufacturer’s protocol (Applied Biosystems, Foster City, CA, USA). We purified sequenced products using Sephadex columns (G.E. Healthcare, Piscataway, NJ, USA) and ran them on an automated Applied Biosystems 3730*xl* capillary sequencer. We edited sequences using Sequencher v4.10.1 (Gene Codes Corporation, Ann Arbor, MI, USA). GenBank accession numbers are provided for all sequences in supplementary S1 Data.

**Table 1:** PCR primers and annealing temperatures used to amplify mitochondrial and nuclear markers in this study.

**Gene**	**Primer**	**Sequence (5'–to–3')**	**PCR steps** ^§^	***T***_A_ **(annealing temperature,°C)**	**Reference**
cyt*b*	L14725	GAYTTGAARAACCAYCGTTG	Single PCR	48	Hrbek *et al*. [106]
	H15982	CCTAGCTTTGGGAGYTAGG	Single PCR	48	Hrbek *et al*. [106]
*cox1*	FISH-F1	TCAACCAACCACAAAGACATTGGCAC	Single PCR	48–49	Ward *et al*. [107]
	FISH-R1	TAGACTTCTGGGTGGCCAAAGAATCA	Single PCR	48–49	Ward *et al*. [107]
*ldh-A*	LDHA6F2	GYGGAGAGCATCSWKAAGAACMTGC	Single PCR	48–49	Quattro & Jones [108]
	LDHA6R*	GCTSAGGAASACCTCRTCCTTCAC	Single PCR	48–49	Quattro & Jones [108]
*RPS7*	1F	TGGCCTCTTCCTTGGCCGTC	1^st^ PCR	52	Chow & Takeyama [109]
	3R	GCCTTCAGGTCAGAGTTCAT	1^st^ PCR	52	Chow & Takeyama [109]
	1F.2	CTCTTCCTTGGCCGTCGTTG	2^nd^ PCR–1	52	Unmack *et al*. [74]
	2R.67	TACCTGGGARATTCCAGACTC	2^nd^ PCR–1	52	Unmack *et al*. [74]
	2F.2.cat	GCCATGTTCAGTACCAGTGC	2^nd^ PCR–2	52	Unmack *et al*. [74]
	3R.10	TCAGAGTTCATCTCCAGCTC	2^nd^ PCR–2	52	Unmack *et al*. [74]
X-*src*	SRC.E7.1F	TGACAGACGTTTGTCCCGTACTGAAGC	1^st^ PCR	52	Peter J. Unmack
	SRC.E10.endR	ATGAGKCGAGCCAGACCGAAATCAGC	1^st^ PCR	52	Peter J. Unmack
	SRC.E8.1F	CTGAAGCCTGGCACCATGTC	2^nd^ PCR	52	Peter J. Unmack
	SRC.E10.end2R	CCGAAATCAGCCACTTTACAMACCAG	2^nd^ PCR	52	Peter J. Unmack
X-*yes*	Yes F1	GAGAGAATGAACTACATCCATAG	1^st^ PCR	52	Peter J. Unmack
	Yes R1	GACCACACGTCTGATTTGATTGTGAA	1^st^ PCR	52	Peter J. Unmack
	Yes F2	GACAACCTGGTCTGTAAGATCGC	2^nd^ PCR	52	Peter J. Unmack
	Yes R2	GATTTGATTGTGAAGCGACCGTACA	2^nd^ PCR	52	Peter J. Unmack
*Glyt*	Glyt_F559	GGACTGTCMAAGATGACCACMT	1^st^ PCR	55	Li *et al*. [110]
	Glyt_R1562	CCCAAGAGGTTCTTGTTRAAGAT	1^st^ PCR	55	Li *et al*. [110]
	Glyt_F577	ACATGGTACCAGTATGGCTTTGT	2^nd^ PCR	62	Li *et al*. [110]
	Glyt_R1464	GTAAGGCATATASGTGTTCTCTCC	2^nd^ PCR	62	Li *et al*. [110]

^§^Single PCR, only one PCR performed; 1^st^ PCR or 2^nd^ PCR, indicates the sequence in a nested set. Note also that a number *n* preceded by a dash in this column (e.g. “–2”) indicates the *n*th second PCR step in a set of nested reactions.

Mitochondrial DNA sequences contained no gaps and were aligned by visual inspection in Sequencher; however, nuclear sequences were aligned in MAFFT v6.850 [75] using the local pair FFTS algorithm with a gap opening penalty of 1.53, a tree rebuilding number of 10, and MAXITERATE = 50. We used PHASE v2.1 [76,77] to determine the most probable pair of alleles for each of the nuclear loci, by resolving heterozygous sites. We ran PHASE in DnaSP v5.10 [78] for 100 iterations with thinning interval = 1 and ‘burn-in’ = 100. We ran three PHASE trials per locus to ensure consistency among phased allelic positions over the output probability threshold, and we used phased alleles in our analyses wherever possible (S1 Appendix).

We collated four datasets used in our analyses. First, we created a ‘full-cyt*b*’ dataset of 941 *Poecilia* sequences by augmenting our database with 68 Mexican cyt*b* sequences (37 haplotypes) from Tobler *et al*. [67] and Palacios *et al*. [68]; this increased our ingroup (with *P*. *sulphuraria*, *P*. *thermalis*, *P*. *butleri*, *P*. *mexicana mexicana*, and *P*. *m*. *limantouri*) and outgroup (*P*. *latipinna* and *P*. *latipunctata*) sampling. Using TCS v1.21 [79], we collapsed identical ingroup cyt*b* sequences into haplotypes, then generated a statistical parsimony network of ingroup haplotype clades (95% connection limit; data not shown) that we used as a basis for selecting individuals to sequence for subsampling at *cox1* and nuclear loci. A second ‘concatenated mtDNA’ dataset was comprised of 171 mtDNA subsamples (*n* = 155 cyt*b* sequences and *n* = 115 *cox1* sequences) spanning all mtDNA major-lineages, taxa, and OTUs that we sampled (S1 Fig). Third, a ‘concatenated nDNA’ dataset contained 50 ingroup samples for up to 5 nuclear loci (S2 Fig). Last, a fourth ‘concatenated mtDNA + nDNA’ dataset contained 80 samples (*n* = 50 ingroup samples and *n* = 30 outgroup samples) sequenced at 6 loci, including the mtDNA locus and up to 5 nuclear loci. We included sequences from [67,68] that formed exclusive mtDNA major-lineages in each dataset, except the concatenated nDNA dataset.

### Neutrality and Recombination

We evaluated the selective neutrality of each mtDNA gene in our analysis using Hudson-Kreitman-Aguadé tests (HKA; [80]) in DnaSP, testing significance using 1000 coalescent simulations. We ran HKA tests using *P*. *caucana* sequences as outgroups, following [55]. We tested each nuclear locus for recombination using six automated tests implemented in RDP3 v3.44 [81], described in S1 Appendix. We also tested for recombination using 1000 coalescent simulations of the minimum number of recombination events (*R*_M_), assuming the empirical per-gene level of recombination estimated in DnaSP. All parameters were simulated in DnaSP given mutation parameter *θ* (= 4*N*_e_μ for autosomal nuclear loci; for mtDNA, *θ* = 2*N*_ef_μ). We considered evidence for recombination in a locus significant by cross-validation if a majority of the seven methods used detected recombination events.

### Gene Tree Analyses and Sequence Divergence

We estimated gene trees for *P*. *sphenops* species complex haplotypes and outgroup sequences in the concatenated mtDNA, concatenated nDNA (overall, and for each locus), and concatenated mtDNA + nDNA datasets using maximum-likelihood (ML) tree searches in GARLI v2.0 [82]. In GARLI, we partitioned the mtDNA data by codon position ({1+2}, 3) and the nDNA into data subsets by gene. We assigned each data subset its best-fit nucleotide substitution model (S2 Table) selected using the decision-theory algorithm DT-ModSel [83], and we unlinked parameters across data subsets. We evaluated nodal support using 500 ML bootstrap pseudoreplicates, considering nodes with bootstrap proportions (BP) ≥70 well supported [84]. We also estimated gene trees, divergence times, and evolutionary parameters (e.g. substitution rates) for each locus using Bayesian inference analyses. To obtain an ultrametric time tree for species delimitation analyses below, we conducted a coalescent-dating analysis of the concatenated mtDNA dataset in BEAST v2.0.2 [85]. We linked tree and clock models but partitioned the data into codon position subsets ({1+2}, 3) and unlinked site parameters across subsets. To ensure convergence, we ran three replicate searches (MCMC = 10^8^, sampled every 4000 generations; burn-in = 10%) using relaxed, uncorrelated lognormal (ULN) molecular clocks. Birth-death tree priors were selected for each run, since this process is well suited for multispecies datasets with varying degrees of lineage divergence. We set uniform priors on ULN clock rates spanning protein-coding mitochondrial gene substitution rates for teleost fishes (‘fish rate’ = 0.017–0.14 × 10^−8^ substitutions/site/yr, per-lineage; refs. in [86,87]). Including *Poecilia* (subgenus *Limia*) outgroups in these analyses provided a calibration point constraining the split between *P*. (*L*.) *domicensis* from Cuba and *P*. (*L*.) *vittata* from Hispaniola to 17–14 million years ago (Ma), based on phylogenetic data [88] and dates for the geological separation of Cuba and Hispaniola, following [55] and references therein. We calibrated this node using a lognormal prior (mean in real space = 1, log standard deviation = 1.25, offset = 14). We used a similar calibration to constrain the tree’s root age to 39.9 Ma with an extended tail (log standard deviation = 2.5), based on the oldest fossil poeciliids available from the Maíz Gordo and Lumbrera formations, Argentina [89]. We also estimated a gene tree for each nuclear locus in BEAST using short runs (MCMC = 20 million, sampled every 1000 generations; burn-in = 10%) specifying ULN clocks and birth-death tree priors. We summarized posterior parameter distributions and ensured that effective sample sizes (ESS) were >200 in Tracer v1.5 [90]. We summarized the posterior distribution of trees from each run by calculating a maximum clade credibility (MCC) tree annotated with median node ages from a sample of 5000 post-burn-in trees in TreeAnnotator v2.0.2 [85].

We estimated evolutionary sequence divergences among major-lineages recovered in our mtDNA gene tree analyses, and species delimitation analyses below, using genetic distances. Mean among-clade *p-*distances were calculated in mega5 [ 91 ] as the number of base differences per site, averaged over all corresponding sequence pairs between groups in the full-cyt*b* dataset. We evaluated variance in the *p*-distances by estimating their standard errors using 500 bootstrap replicates. For comparison, we also estimated divergence between each of these ingroup clades and the two outgroup ‘sail-fin’ molly species (*P*. *latipinna* and *P*. *latipunctata*). We archived our sequence alignments and ML and Bayesian gene tree results in Dryad (doi:10.5061/dryad.m1g3v).

### Coalescent-based Species Delimitation

We delimited species in the *P*. *sphenops* species complex using a multi-tiered Bayesian approach involving an initial species discovery step, followed by species validation. We base this ‘chimeric’ approach on previous studies [21,43,49], and recognition that the accuracy of species validation methods relies critically on accurate *a priori* species assignments, as well as guide trees (see below). First, we used the general mixed Yule-coalescent model (GMYC; [30,34]) to assign individuals to species and develop a preliminary set of hypothesized species limits. The GMYC identifies the transition point between speciational and coalescent branching processes on an ultrametric time tree derived from single-locus data [30]. Importantly, the model makes standard coalescent assumptions (neutrality, constant population size and mutation rate, no extinction) but no *a priori* assumptions about species boundaries. We used the Bayesian GMYC model implemented in the R package bGMYC [34] to discover species in the MCC tree from the concatenated mtDNA matrix. By accounting for phylogenetic error and allowing multiple threshold points across the tree (*cf*. [31]), bGMYC overcomes two main shortcomings of Pons *et al*.’s [30] original ML model. As bGMYC is prone to over-split trees containing identical alleles (i.e. zero-length branches) into species [34], we dropped any zero-length tips from the MCC tree prior to analyses, then ran bGMYC using the single- and multiple-threshold models. For conservativeness and increased statistical power at species discovery (lower false positive, or Type I error, rate), we interpreted results as significant at a modified α = 0.10 level. Tree depth heavily influences GMYC results so that transition points may not be detectable when speciation and coalescence rates are similar [34]. Thus, we checked speciation and coalescence rates in the MCC tree empirically using the python script “PTP.py” [92]. We also tested the assumption that the MCC tree contained two classes of branching processes, by performing likelihood-ratio tests comparing single- and multiple-threshold ML GMYC models against null models with one branching process (implying either that all tips are species, or the data represent a single species) in the R package SPLITS v2 [93].

Next, we used two Bayesian methods to validate and better infer the evolutionary history of the GMYC-delimited species: we estimated a multilocus species tree and divergence times and then independently tested the validity of each (originally mtDNA-inferred) species by estimating its Bayesian posterior probability (PP) on the species tree using only nuclear loci. We inferred the species tree and divergence times for the delimited species using the multispecies coalescent *BEAST method [39] implemented in BEAST. We ran *BEAST using all loci in the concatenated mtDNA + nDNA dataset and assigning individual sequences to 25 species, including delimited ingroup species (with at least two sequences per species) plus 15 outgroup taxa (see Results, S1 Appendix). Outgroups permitted setting two calibration points on the same nodes using lognormal priors identical to those in the calibrated BEAST analyses above. We ran *BEAST for five runs of 200 million generations each, sampling every 5000 generations, using Yule tree priors. Log files from each run were combined using LogCombiner v2.0.2 [85] and we visually checked the final log for proper MCMC convergence and mixing and ensured that ESS scores were >200 in Tracer. Tree files were reduced in size and combined before a MCC tree was computed from 5000 post-burn-in trees in TreeAnnotator.

We tested the validity of the GMYC-delimited species using the Bayesian species delimitation method implemented in BP&P v2.1 [25], which uses a reverse-jump MCMC (rjMCMC) algorithm to generate marginal posterior probabilities for species-delimitation models using multilocus genetic data. BP&P accounts for gene tree variance and ILS, and calculates mutation-scaled population size (*θ*) and divergence time (*τ*) estimates. BP&P also assumes that no gene flow occurs following speciation, analogous to the biological species criterion of Mayr [94]. We ran BP&P on the concatenated mtDNA + nDNA dataset fully partitioned by gene, using the *BEAST species tree as a guide tree, and specifying a Dirichlet distribution (*α* = 2) to account for variation in mutation rates among loci. Because BP&P is sensitive to the choice of priors [95], we assessed the impact of prior specification on our results by conducting runs using three different combinations of gamma-distributed priors for ancestral *θ* and root age (*τ*_0_) [43]: large ancestral populations and deep divergences, *θ ~* G(1, 10) and *τ*_0_ ~ G(1, 10); small ancestral populations and shallow divergences, *θ ~* G(2, 2000) and *τ*_0_ ~ G(2, 2000); and a highly conservative prior with large ancestral populations and recent divergences, *θ ~* G(1, 10) and *τ*_0_ ~ G(2, 2000). We made three replicate runs (rjMCMC = 10^6^; burn-in = 25,000) of each prior combination using algorithm 0 (default fine-tuning parameter, *ε =* 15) and algorithm 1 (*α* = 2, *m* = 1). We conservatively accepted daughter lineages from nodes with speciation probabilities ≥0.95 across all three priors as strongly supported species.

### Hybridization *Versus* Incomplete Lineage Sorting

Our analyses indicated several points of discordance between gene trees derived from different loci (see Results), which is often caused by hybridization-mediated introgression, or ILS arising from the retention of ancestral polymorphisms [38,96]. Whereas these two confounding genetic processes are difficult to tease apart, a recent molecular study of the *P*. *sphenops* species complex by Alda *et al*. [55] inferred hybridization at the nuclear *RPS7* locus between two pairs of lineages in the complex that we also sampled in this study, *P*. *catemaconis*-*P*. *sphenops* and *P*. *mexicana*-“*gillii*” sp. 2, but no evidence for mtDNA hybridization. Thus, available data suggest that incongruences we observed among gene trees, particularly between mtDNA and nDNA gene trees, may be due to introgression in the nuclear genome. We conducted multiple analyses to determine whether the source of gene tree discordance was more likely due to gene flow *versus* ILS. First, we estimated the degree of exclusive ancestry of individuals of species as quantified by the genealogical sorting index (*gsi*; [97]). The *gsi* spans values normalized to the interval [0, 1], with 1 indicating monophyly, <1 indicating paraphyly, and 0 indicating non-exclusive ancestry in relation to other sampled species. We calculated *gsi* for delimited species based on ML gene trees derived from the concatenated mtDNA dataset, each nuclear locus, and the concatenated mtDNA + nDNA dataset. We also calculated an ‘ensemble’ *gsi* statistic (*gsi*_*T*_) as the weighted sum of *gsi* across all five nuclear gene trees. Cummings *et al*. [97] showed that, by integrating across multiple loci, *gsi*_*T*_ has sufficient power to detect significant genealogical divergence well before monophyly is reached, even using small numbers of loci. Analyses were run on the *gsi* web server (http://www.genealogicalsorting.org) while assigning individuals to delimited species, and testing significance using 10^4^ permutations.

Second, we used Joly *et al*.’s [96] method for detecting hybridization from species trees, as implemented in JML v1.0.2 [98]. JML uses posterior predictive checking to detect hybridization by testing the fit of a null model with no hybridization (but ILS) to sequence data, through simulations conducted on a posterior sample of species trees from *BEAST (thereby accounting for phylogenetic error). We supplied JML with 1000 post-burn-in species trees from a *BEAST analysis consisting of five independent runs similar to those above (assigning individuals to delimited species, MCMC = 200 million, burn-in = 10%, birth-death tree priors, and a constant multispecies coalescent population function) but using ingroup samples. We then simulated gene trees and DNA sequence datasets on each species tree under a neutral coalescent model with no migration. For simulations, we specified ML estimates of model parameters from GARLI, evolutionary rates estimated in *BEAST, and appropriate heredity scalars (2 for nDNA, 0.5 for mtDNA) for each locus. We ran separate simulations drawing on ingroup mtDNA sequences from the concatenated mtDNA + nDNA dataset, plus the three nDNA loci with the most sampling (*ldh-A*, *RPS7*, and X-*src*). For each simulated dataset, we computed distributions of the minimum pairwise sequence distance between sequences of two species (*minDist*), a good predictor of hybridization events [96]. We evaluated fit of the ILS model (i.e. adequacy of *BEAST model fit to the data) by comparing *minDist* for the observed data to that of the simulated datasets, to calculate the probability that observed distances were due to hybridization. Using a one-tailed test, we rejected the ILS model at the α = 0.05 level in favor of hybridization being the most likely explanation for observed DNA polymorphism patterns between species pairs [98]. For nDNA loci, we only considered significant results meaningful for taxa with observed sequence data, rather than simulated data alone (e.g. the case of clade 7), because while observed sequences are optional for JML an observed pair of aligned sequences is required to calculate exact probabilities of *minDist* values.

## Results

### Neutrality and Recombination

Based on HKA tests, DNA polymorphism levels in the mtDNA data were consistent with expectations of neutral evolution, which was assumed in each of our analyses (*P* > 0.05; details in S1 Appendix). Likewise, an outstanding majority of tests (91.4%) recovered no evidence for recombination in any of the nuclear loci analyzed (S1 Appendix): six tests of each of five loci in RDP3 inferred a total of only three recombination signals (all in X*-yes*), and coalescent simulations showed no evidence of recombination based on *R*_M_ values (*P* > 0.05; S1 Appendix).

### Gene Tree Analyses and Sequence Divergence

**Fig 2:**
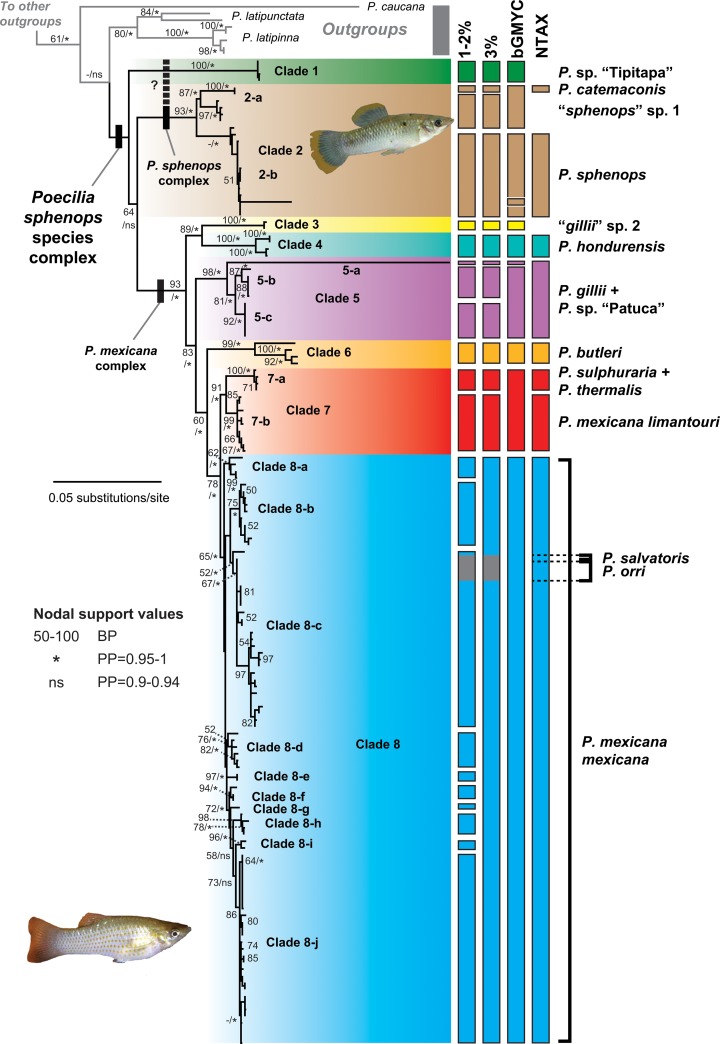
Results of bGMYC analysis for developing preliminary species delimitation hypotheses. Results presented are based on the concatenated mtDNA (cyt*b*, *cox1*, serine tRNA) dataset and represented on the gene tree resulting from maximum-likelihood (ML) analysis in GARLI. Nodal support values are ML bootstrap proportions (BP; ≥50%)/Bayesian posterior probabilities (PP; ≥0.95). Colored bars to the right of the phylogeny represent hypothesized species groupings based on ≥0.9 Bayesian posterior probability of conspecificity (calculated from Bayesian MCMC analysis of 100 post-burn-in trees from the concatenated mtDNA BEAST analysis), compared with bars demarcating clades meeting genetic distance thresholds (1–2%, 3%) and nominal taxonomy (NTAX).

The concatenated mtDNA dataset consisted of 1770 nucleotide base pairs (bp), including a 1086 bp fragment of cyt*b* and 684 bp of the partial *cox1* gene and flanking serine tRNA (S3 Table). The ML gene tree derived from this dataset had a ln *L* of −12419.3689 and generally recovered well supported relationships among ingroup lineages, with BP > 70% for most tip clades and internal nodes (Fig 2). However, mtDNA lineages in the gene tree provided a variable fit to nominal taxonomy and currently recognized OTUs [51,55]. Haplotypes of *P*. *butleri*, *P*. *gillii*, “*gillii*” sp. 2, *P*. *hondurensis*, and *P*. sp. “Patuca” were recovered as highly supported monophyletic groups, and relationships among these lineages received moderate to high bootstrap support. Members of the *P*. *sphenops* complex *sensu stricto*, including *P*. *catemaconis*, *P*. *sphenops*, and “*sphenops*” sp. 1, were also monophyletic, although *P*. *sphenops* monophyly was poorly supported. By contrast, *P*. *mexicana* was polyphyletic, with samples from Río Tipitapa, Nicaragua between Lake Managua and Lake Nicaragua (Fig 1) recovered in a monophyletic group at the base of the complex *sensu lato*; and *P*. *orri* and *P*. *salvatoris* were each paraphyletic, nested within the principal *P*. *mexicana* clade. The position of the Tipitapa lineage was poorly resolved by mtDNA, and its sister relationship to all other *P*. *sphenops* species complex lineages received marginal support, yet given its genetic distinctiveness we refer to this *P*. *mexicana*-like lineage as a ‘candidate species’, *P*. sp. “Tipitapa”. We also recovered *P*. *thermalis* in a clade containing *P*. *sulphuraria*; however, these taxa shared identical cyt*b* haplotypes. For convenience of presentation and discussion, we identified eight mtDNA major-lineages (clades 1–8) in the gene tree differentiated by ≥2% mean among-clade *p*-distances (range 2.3–9.9%; Table 2), which we visualized with distinct colors. We also identified 17 exclusive, moderate to strongly supported ‘subclades’ contained within these major-lineages (2-a to 8-j) in the mtDNA gene tree.

**Table 2:** Mean pairwise genetic distances among 10 clades accepted as preliminary species hypotheses based on Bayesian general mixed Yule-coalescent (GMYC) results in Fig 2.

	**Clade 1**	**Clade 2-a**	**Clade 2-b**	**Clade 3**	**Clade 4**	**Clade 5-a**	**Clade 5-b**	**Clade 6**	**Clade 7**	**Clade 8**
**Clade 1**	–	0.0080	0.0081	0.0087	0.0089	0.0087	0.0087	0.0082	0.0082	0.0082
**Clade 2-a**	0.085	–	0.0044	0.0069	0.0072	0.0071	0.0070	0.0072	0.0072	0.0068
**Clade 2-b**	0.082	0.031	–	0.0072	0.0075	0.0075	0.0076	0.0072	0.0074	0.0071
**Clade 3**	0.091	0.070	0.070	–	0.0065	0.0068	0.0066	0.0068	0.0067	0.0064
**Clade 4**	0.086	0.070	0.071	0.052	–	0.0064	0.0064	0.0066	0.0062	0.0057
**Clade 5-a**	0.094	0.071	0.073	0.060	0.057	–	0.0030	0.0062	0.0050	0.0051
**Clade 5-b**	0.096	0.074	0.077	0.060	0.058	0.013	–	0.0062	0.0053	0.0051
**Clade 6**	0.099	0.075	0.074	0.061	0.059	0.055	0.059	–	0.0054	0.0052
**Clade 7**	0.090	0.078	0.075	0.061	0.051	0.041	0.046	0.049	–	0.0031
**Clade 8**	0.089	0.071	0.072	0.056	0.048	0.040	0.043	0.046	0.023	–

Below the diagonal, mean among-clade *p*-distances based on the full-cyt*b* sequence database; above the diagonal, corresponding standard error values.

**Fig 3:**
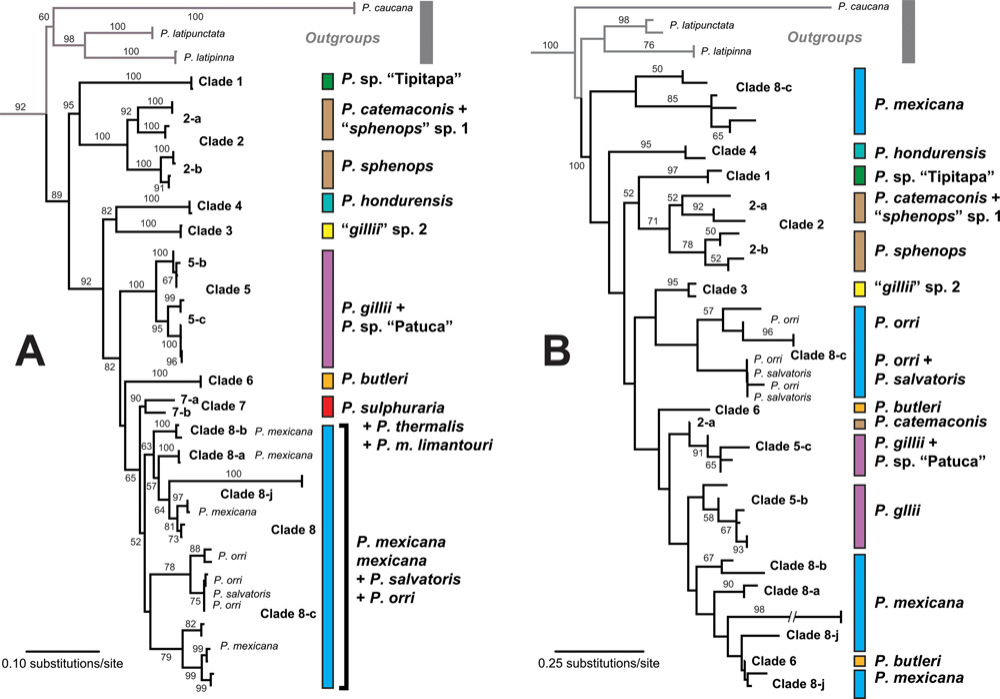
Gene trees derived from maximum-likelihood analyses of the concatenated mtDNA + nDNA dataset (A) and the concatenated nDNA dataset (B) in GARLI. Numbers along branches indicate the level of nodal support from ML bootstrap proportions (BP) ≥50%. Clade names at tips and colored bars representing delimited/nominal species correspond to those shown in Fig 2.

The BEAST relaxed clock analysis of the concatenated mtDNA dataset converged on a mean *L* of −12,590.73 and had good sampling properties (e.g. ESS > 316). From this run, we generated a MCC time tree (highest log clade credibility = −139.6855; S3A Fig) that recovered ingroup relationships identical to the mtDNA ML gene tree, but with higher nodal support values (e.g. PP = 0.95–1 for most ingroup tip clades and internal nodes; Fig 2). Unlike the mtDNA ML gene tree, however, we recovered one Lake Nicaragua tributary sample (172554) sister to other clade 2 samples with strong support (S3A Fig). Nuclear genes in the concatenated nDNA dataset (3484 bp), and also in the concatenated mtDNA + nDNA dataset, were on average 685 bp long (range 191–967 bp), and averaged 59.6 variable characters, 45 parsimony informative characters, and 0.017 overall mean *d* based on *p*-distances (S3 Table). Phylogenetic structuring in the ML gene tree derived from the concatenated mtDNA + nDNA dataset (ln *L* = −21,978.2011) mirrored relationships recovered in the concatenated mtDNA gene trees, except *P*. sp. “Tipitapa” was recovered sister to the *P*. *sphenops* complex *sensu stricto* (clade 2) with high support, clade 8-j was recovered in a monophyletic group with representatives of clades 8-a and 8-b, and while phylogeographical sub-structuring in clade 8-c was well supported the monophyly of clade 8-c itself was poorly supported (Fig 3A). The concatenated nDNA gene tree was relatively less resolved than the other gene trees but also placed *P*. sp. “Tipitapa” sister to clade 2 with moderate support, and strongly supported the monophyly of clades 1–4 (Fig 3B). Evaluating each nuclear locus separately also indicated lower resolution, and along with varying degrees of genetic variation we observed differing degrees of species monophyly at different loci (S4 Fig; S3 Table). Although different methods and datasets varied in the levels of support assigned to nodes in the tree, all of the analyses essentially identified the same major-lineages and recovered *P*. *orri*, *P*. *salvatoris*, and *P*. *thermalis* as paraphyletic (Fig 2 and S2 and S3 Figs).

### Coalescent-based Species Delimitation

Separate bGMYC runs specifying different models gave very similar preliminary hypotheses of species boundaries, although the multiple-threshold model estimated finer groupings leading to slightly higher species diversity than the single-threshold model. Running the single-threshold model gave a pattern of 11 species that met our criteria (S5A Fig), eight of which corresponded to mtDNA major-lineages identified using the gene tree and *p*-distances (Fig 2). Similarly, the multiple-threshold model supported 14 species (S5B Fig). Both models assigned species status to the single tip sample 172554 from clade 2 and sample 23082 from clade 5; however, such low allele sampling is non-optimal for bGMYC, and sample 172554 was consistently recovered within clade 2-b in the mtDNA ML gene tree analysis with strong support, so we conservatively considered only the subclades in these groups/clades defined by multiple individuals as potential species (subclades 2-a and 2-b; *cf*. [21]). Thus, we accepted a more conservative number and arrangement of clusters of 10 species with multiple individuals from the single-threshold bGMYC analysis as our preliminary species delimitation hypothesis. Rate calculations indicated that the GMYC results were unlikely to be confounded by proximal speciation and coalescence rates, which diverged widely (speciation rate per substitution, λ_*s*_ = 19.64; coalescent rate per substitution, λ_*c*_ = 508.67). Moreover, likelihood-ratio tests performed in SPLITS confirmed that the two classes of branching processes assumed in the model were present in the tree (single-threshold test: null ln *L* = 637.54, max. ln *L* = 644.70, likelihood ratio = 14.32, *P* < 0.01; multiple-threshold test: null ln *L* = 637.54, max. ln *L* = 646.08, likelihood ratio = 16.93, *P* < 0.01).

The relaxed clock *BEAST species tree (mean *L* = −21,865.97, ESS = 1,382.69) inferred relationships among the *P*. *sphenops* species complex that were identical to those recovered in the concatenated mtDNA + nDNA ML tree, placing a strongly-supported monophyletic group containing clades 1 and 2 sister to all other members of the *P*. *sphenops* species complex *sensu lato* with strong support (S3B Fig). Predictably, subsamples representing phylogeographic structuring within clades 2 and 8 were recovered as monophyletic. However, the monophyletic group containing clades 3–8 differed from the concatenated mtDNA gene tree in placing clade 6 sister to clades 5 + 7–8, rather than clade 5 sister to clades 6–8 (as in Figs 2 and 3A), although relationships among these clades were poorly supported. Based on the time to the most recent common ancestor (*t*_MRCA_) estimated by *BEAST for the stem node splitting a *P*. *caucana* + ‘short-fin’ mollies clade and the ingroup, we inferred a maximally early-mid Miocene origin for the ancestral ingroup population in Central America [median age = 16.4 Ma, 95% highest posterior density (HPD) = 23.2–11.1]. Moreover, the ingroup *t*_MRCA_ indicated the diversification of the *P*. *sphenops* species complex *sensu lato* most likely began 17. 6–8.1 Ma (median age = 12.2) in the Miocene and continued to the present. *Poecilia* sp. “Tipitapa” was the oldest species (median age = 9.2 Ma, 95% HPD = 14.4–5.4), whereas *P*. *sphenops* complex clades 2-a and 2-b were the youngest delimited species, with a Plio-Pleistocene *t*_MRCA_ (median age = 2.4 Ma, 95% HPD = 4.7–0.54).

**Fig 4:**
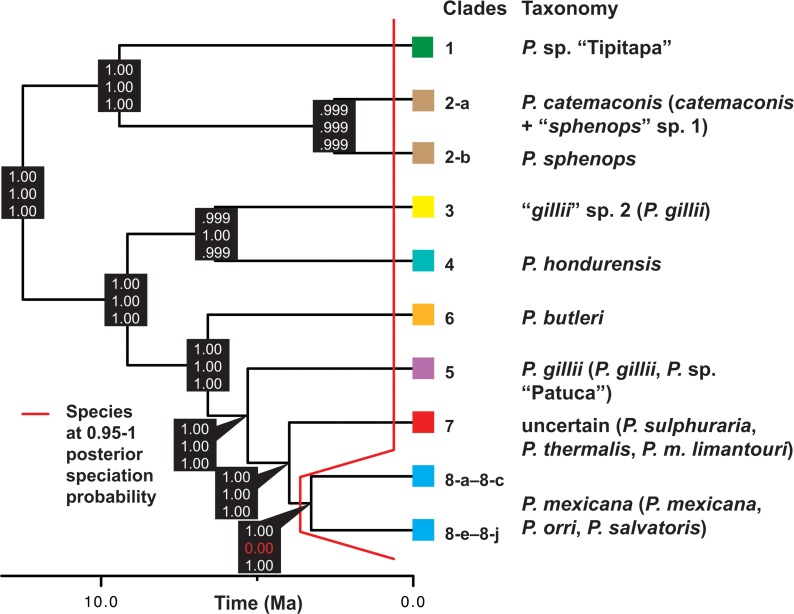
Species tree inferred for the *P*. *sphenops* species complex showing speciation probabilities for each node. Bayesian speciation probabilities are posterior probabilities that a node is fully bifurcating and are shown for each node under each combination of priors in BP&P (top, large ancestral *θ* and deep root divergence, *τ*_0_; middle, small ancestral *θ* and shallow *τ*_0_; bottom, large ancestral *θ* and shallow *τ*_0_). The red line distinguishes between species that were strongly supported (PP ≥ 0.95) using all three arbitrary prior combinations, and those with non-significant speciation probabilities. Results are presented for algorithm 1 runs and species that we could evaluate in BP&P. S4 Fig shows speciation probabilities estimated using BP&P algorithm 0.

Running BP&P with algorithm 1 under priors reflecting different historical scenarios strongly supported each of the 9 delimited species examined with high speciation probabilities (Fig 4). However, the clade 8 crown node containing phylogeographical structuring between subclades 8-a–8-c and subclades 8-e–8-j received significant support from the models with large and small ancestral sizes and deep divergences (PP = 1), but no support from the small ancestral size, shallow divergence model (PP = 0). Quantitatively and qualitatively similar results were obtained in identical runs using algorithm 0 (S6 Fig). Given uncertainty in the internal nodes of our species tree, we also ran BP&P on the concatenated mtDNA + nDNA ML gene tree topology, and this yielded near-identical results. Thus, multilocus Bayesian species delimitation based on the present sampling strongly supports recognizing clades 1, 2-a, 2-b, and 3–8 as distinct species with 95% Bayesian posterior probability, but indicates that phylogeographical lineages within clade 8 receives substantial but not definitive support and cannot be treated as distinct species. Clade 7 was only evaluated in BP&P using mtDNA sequences from [67,68]; however, its monophyly and significant nodal support in the ML and Bayesian gene trees (Figs 2 and 3), high Bayesian PP in the GMYC results, and the mtDNA *gsi* results below, indicate that clade 7 would likely have been strongly supported as a distinct species in BP&P had nDNA loci been available for this lineage.

### Hybridization *Versus* Incomplete Lineage Sorting

Permutation tests of the *gsi* calculated from the mtDNA ML gene tree in Fig 2 supported each bGMYC-delimited species as a monophyletic lineage in relation to other delimited species, with mostly complete lineage sorting (*P* < 0.001; Table 3). Likewise, *gsi* tests supported all delimited species monophyly in the concatenated mtDNA + nDNA gene tree. We also detected significant genealogical divergence and sorting at different nDNA loci for most species, despite a lack of monophyly (18/27, or 67% of cases; Table 3). However, *gsi* values expectedly fluctuated across nDNA loci, with values for loci with more variable characters tending to be higher, and with delimited species being consistently significantly sorted at *RPS7* and X-*src* but less consistently so at other loci (Tables 2 and 3). Still, all taxa with nuclear data had significant ensemble *gsi* scores (mean *gsi*_*T*_ = 0.384) across the nuclear gene trees (*P* < 0.05; Table 3).

**Table 3:** Genealogical sorting index (*gsi*) scores and significance test results for GMYC-delimited species of the *Poecilia sphenops* species complex.

		**nDNA loci**						
**Delimited species**	**Concatenated mtDNA**	***Ldh-A***	***RPS7***	**X-*src***	**X-*yes***	***Glyt***	**Ensemble score (*gsi***_*T*_ **)**	**Concatenated mtDNA + nDNA**
Clade 1, *P*. sp. “Tipitapa”	1**	0.039ns	0.488*	0.488*	–	–	0.203*	1*
Clade 2-b	1**	0.029ns	1**	1*	–	–	0.406*	1**
Clade 2-c, “*sphenops*” sp. 1	1**	0.083ns	1**	0.463*	0.472ns	1*	0.603**	1**
Clade 3, “*gillii*” sp. 2	1**	0.035ns	0.488*	1**	–	–	0.305*	1*
Clade 4, *P*. *hondurensis*	1**	0.024ns	1*	0.488*	–	–	0.302*	1*
Clade 5, *P*. *gillii*	1**	0.180ns	1**	0.551**	0.367*	0.250ns	0.470**	1**
Clade 6, *P*. *butleri*	1**	–	–	–	–	–	–	1*
Clade 7, “*limantouri*” clade	1**	–	–	–	–	–	–	1*
Clade 8, *P*. *mexicana* clade	1**	0.279*	0.688**	0.472**	0.336*	0.205ns	0.396**	1**

*P < 0.05;

**P < 0.001; ns, not significant

We detected no instances of ingroup mtDNA introgression based on 1000 coalescent simulations in JML. Thus, we conclude that post-speciation hybridization at mtDNA is unlikely, and that the multispecies coalescent model in *BEAST provides a good fit to the mtDNA data. Therefore, the mtDNA are also consistent with assumptions of BP&P [25]. By contrast, JML simulations detected introgressed sequences at each of the three nuclear loci examined (S1 Appendix). In particular, we consistently detected introgressed sequences between the *P*. *butleri*-*P*. *catemaconis/sphenops* (clade 2-a) species pairs across all three loci, based on significant departures of observed *minDist* values from the posterior predictive distributions (*ldh-A*, *P* = 0.001; *RPS7*, *P* = 0.001; and X-*src*, *P* = 0.001; S1 Appendix). These findings suggest that the *BEAST model provides an inadequate fit to these three nuclear markers because it assumes that all gene tree discordance is due to ILS. Overall, our JML results indicate that the probability of obtaining para-/polyphyletic nDNA gene trees but monophyletic mtDNA gene trees is high, and that gene tree discordances observed in this study have likely resulted from hybridization instead of ILS in the nuclear genome. In particular, the low PP for the placement of *P*. *butleri* in the species tree (S3 Fig) seems likely due to hybridization.

## Discussion

A growing number of empirical studies suggest that newly developed coalescent-based species delimitation methods [29] provide effective tools for delimiting species in morphologically conserved groups with cryptic species, using independent genetic loci [9,20,21,43,46–48]. Indeed, these methods are recommended to overcome the limited utility of morphology to delimit species in these systems, e.g. few diagnostic characters distinguishing species [21,43,46]. One advantage of coalescent-based methods is that, whereas earlier species delimitation approaches based solely on phylogenetic criteria (‘phylogenetic species concepts’) required strict assumptions of monophyly and fixed allelic differences at one or more genetic loci (reviewed by [12]), coalescent species delimitation relaxes these constraints, given such patterns are not expected in multilocus datasets [26,38,99]. Thus, coalescent-based species delimitation methods can identify independently evolving lineages representing distinct species through probabilistic tests of alternative speciation hypotheses (e.g. different resolutions of species tree branches) while allowing for gene tree discordance and ILS (reviewed by [29]). Using a “chimeric approach” [46] combining coalescent methods for single-locus species discovery without assuming species boundaries *a priori* (i.e. Bayesian GMYC modeling; [34]), and Bayesian species delimitation using multiple independent loci (i.e. BP&P; [25]), we set out to delimit species and infer evolutionary relationships in a morphologically conserved group of Central American freshwater fishes, the *Poecilia sphenops* species complex [50,51]. Other studies have used similar approaches to delimit terrestrial and freshwater species, and served as bases for new species descriptions in several cases [9,20,43,46,47,49]. Yet ours is the first attempt to resolve taxonomic uncertainties in the Central American freshwater biota using coalescent-based species delimitation. Overall, our results provide compelling evidence for incongruence between genetically delimited species and nominal taxonomy indicating diversity is underestimated and overestimated in different lineages of the *P*. *sphenops* species complex, with important implications for taxonomy and conservation.

### Species Delimitation in the *P*. *sphenops* Species Complex

Many previous systematic studies of poeciliid livebearing fishes, and of the *P*. *sphenops* species complex in particular, have relied solely on classical morphology [50,51,59,60,63,66,100,101]. This has imposed an important limitation on studies of *Poecilia*, given the “confusingly variable” nature of morphology in the *P*. *sphenops* species complex [50], and that fishes in genus *Poecilia* (particularly subgenus *Mollienesia*) may exhibit ample intraspecific variation to swamp interspecific variation [59,60]. Indeed, after studying *Poecilia* including members of the *P*. *sphenops* species complex, Rivas [60] concluded, “there is considerable variation in [morphometric] characters individually, ontogenetically, seasonally, geographically, and environmentally and, therefore, they are of little or no value in distinguishing species” (our clarification in brackets). Also, very few meristic or external morphological characters are useful for diagnosing species in the *P*. *sphenops* species complex, except a handful of characters related to inner jaw tooth dentition, fin-ray counts, and preorbital head pores [50,59,100,101]. Perhaps not surprisingly, morphology-based taxonomy has been extremely confused in the group, with different authors synonymizing up to 34 taxa into *P*. *sphenops* [63] at one extreme, and recognizing at least six subspecies between *P*. *mexicana* [66] and *P*. *gillii* [59] at another. Aside from destabilizing taxonomy in the group, earlier morphological studies also suffered drawbacks of limited spatial sampling, and restricted taxonomic and phylogenetic perspectives focused on one species or species group e.g. [50,70].

Our results from applying coalescent models to genetic data from an extensive geographical sample of 8 of 13 species, one subspecies, and 2/2 molecular OTUs previously recognized in the *P*. *sphenops* species complex (Fig 1; S1 Table) strongly support at least 9 lineages as distinct ‘species’. These include: (1) *P*. *butleri* (clade 6); (2) *P*. *catemaconis*/*sphenops* (including *P*. *catemaconis* and “*sphenops*” sp. 1 samples, clade 2-a); (3) *P*. *gillii* (clade 5-b and 5-c); (4) *P*. *hondurensis* (clade 4); (5) *P*. *mexicana* (clade 8); (6) *P*. *sphenops* (clade 2-b); (7) clade 7, including multiple Mexican taxa; (8) the *P*. sp. “Tipitapa” lineage (clade 1), discovered in this study and identified in the field as *P*. *mexicana*; and (9) the “*gillii*” sp. 2 lineage (clade 3), initially discovered and identified in the field as *P*. *gillii* by Alda *et al*. [55]. Fig 4 summarizes the placement and inferred taxonomy of each lineage in the species tree, and Fig 1 provides a map of each lineage’s distribution in a regional context. Each species delimited by our full analysis is supported by multiple lines of evidence, including substantial nodal support in gene trees from the mtDNA or combined analyses, high Bayesian posterior probabilities (PP = 0.9–1) of conspecificity during bGMYC modeling, and high Bayesian speciation probabilities (PP = 0.95–1) in coalescent analyses using BP&P (Figs 2, 3 and 4; S1 and S4 Figs). Moreover, putative species are distinct from one another by ≥2% and more frequently ≥3% mean pairwise mtDNA genetic distances (Table 2). That said, the ‘cryptic’ candidate species in clades 1 and 3 are highly distinct, being the only taxa except their sister lineages (clades 2 and 4) that are both deeply diverged from other lineages by ≥5% mtDNA genetic distances (Table 2) and strongly supported as monophyletic in all gene tree and species tree analyses (Figs 2, 3 and 4). In light of this, our findings demonstrate that species-level diversity within the *P*. *sphenops* species complex is underestimated by at least ~15%, relative to the 13 currently described species (S1 Table).

However, we suspect that current diversity within the *P*. *sphenops* species complex is underrepresented by our results, most likely in clades 2-a, 5, and 7. The two lineages lumped into clade 2-a, two lineages in clade 5 (*P*. sp. “Patuca” in 5-b, and 5-c), and two lineages in clade 7 (subclades 7-a and 7-b) respectively diverged from one another fairly recently ~0.86, ~1.14, and ~0.71 thousand years ago during the early-mid Pleistocene (S3 Fig). Given recent lineage divergences may cause Bayesian GMYC modeling to undersplit data into species (discussed below), bGMYC may have generated invalid species designations by lumping tips in into one species in these cases (Fig 2). Clearly, resolving taxonomy in these clades will require additional sampling and analyses of multiple nuclear loci, and a coalescent approach similar to ours is recommended. Our ability to draw conclusions about clade 7 seems particularly limited, as we could not obtain analogous nuclear sequences for samples from Tobler *et al*. [67] and Palacios *et al*. [68].

We have shown that earlier morphological treatments underestimated species-level diversity in the *P*. *sphenops* species complex and particularly within *P*. *mexicana* and *P*. *gillii* e.g. [59,102]. By contrast, our finding that *P*. *orri* and *P*. *salvatoris* are paraphyletic with respect to *P*. *mexicana*, nested within a larger clade otherwise exclusively comprised of *P*. *mexicana* in the mtDNA gene trees (Fig 2 and S1 Fig), suggests nominal taxonomy likely overestimates diversity in clade 8, possibly by up to ~15%. *Poecilia orri* and *P*. *salvatoris* were recovered in a well-supported clade in the concatenated mtDNA + nDNA gene tree (Fig 3A), but *P*. *salvatoris* was nested within *P*. *orri* sequences in this clade, and neither of these species was reciprocally monophyletic in our mtDNA or nDNA gene trees (Figs 2 and 3B). Thus, one or both of these taxa may not constitute distinct species, and this is also supported by the fact that neither taxon was recovered as a distinct species during our species discovery analyses. In fact, bGMYC gave *P*. *orri* and *P*. *salvatoris* samples 95% Bayesian posterior probabilities of conspecificity with *P*. *mexicana* (S5 Fig). Therefore, we suggest that a formal taxonomic revision examining morphological and genetic data be undertaken to determine the status of these taxa.

### Combining Species Discovery and Validation: Limitations and Sampling Considerations

Through our use of a “chimeric approach” [46] to coalescent-based species delimitation combining species discovery and validation methods, this study highlights key interactions between phylogenetic and statistical population genetic (coalescent) analyses typically integrated during such analyses [20,21,43]. Such integration is essential for statistically evaluating evolutionary patterns and processes at the species boundary, the interface between micro- and macroevolution [30]. The particular combination of developing preliminary species delimitation hypotheses through single-locus GMYC modeling, then testing these using multilocus Bayesian species tree and species validation analyses herein also has several strengths. For example, it accounts for gene tree discordance using the multispecies coalescent [39], avoids confounding gene trees with species trees, and also objectively arrives at *a priori* species assignments using coalescent methods implemented *before* conducting separate species validation analyses in BP&P [43,46]. Still, the multiple steps of such chimeric approaches are, overall, subject to several potential limitations, the most important of which we discuss below.

First, uncertainty associated with the topology and branch lengths of ultrametric phylogenies supplied for GMYC modeling can be high because trees are usually derived from single-locus mtDNA datasets. Thus, running GMYC models on a single phylogenetic point estimate could yield inaccurate results, leading to erroneous preliminary hypotheses of species limits [30,34]. Despite this, we consider our GMYC results reasonably accurate, because bGMYC accounts for phylogenetic and modeling error by integrating over uncertainty in the parameters using Bayesian MCMC simulations. It is also important to note that we supplied bGMYC with a valid ultrametric MCC tree generated from a coalescent-dating analysis in BEAST using appropriate priors, including biogeographic and fossil calibration points (S1 and S3 Figs). And our results seem unlikely to reflect confounding effects of branch length uncertainty: analyzing the concatenated mtDNA gene tree (Fig 2) using a Bayesian application of a method, PTP [92], similar to GMYC but analyzing substitution patterns along gene trees with non-ultrametric branches gave species delimitations comparable to our bGMYC results (unpublished data; details in S1 Appendix). This demonstrates that our mtDNA data are robust to the varying assumptions and quantitative approaches of different species discovery methods [30].

Second, recent lineage divergences are also problematic for Bayesian GMYC inference because they induce greater uncertainty into the model and are more likely to occur more recently than inferred threshold points [34]. In our study, the fact that multiple genetic lineages in clades 2-a, 5 and 7 were relatively recently diverged, more so than delimited species, suggests this situation may have caused bGMYC to inaccurately lump these lineages together. This would mean that bGMYC effectively treated actual species-level diversity as intraspecific genetic structuring in these clades. More sampling is necessary to test this hypothesis; however, it may be unrealistic to expect the youngest of these Pleistocene-evolved lineages to fare well in subsequent multilocus validation in BP&P: such recently evolved lineages may not have accumulated enough mutational differences to have high speciation probabilities.

Three additional limitations arise because Bayesian species delimitation using coalescent analyses in BP&P is subject to misspecifications of species limits, guide tree relationships, and model priors [43,95]. Due to the difficulty of confidently establishing species limits *a priori* in non-adaptive radiations with uncertain taxonomy such as the *P*. *sphenops* species complex, it is essential that species discovery analyses used to set up BP&P runs be conducted as rigorously as possible [21]. Whereas, as noted above, we feel our bGMYC results are robust, we do not know whether a multilocus species discovery step, e.g. employing Bayesian assignment tests as per [20,43], would have improved our initial hypotheses of species limits. However, our BP&P results do not seem susceptible to misspecifications of the guide tree or model priors. This is supported by the fact that running BP&P on the species tree as well as the topology from the concatenated mtDNA + nDNA gene tree (unpublished data) gave similar results, and that we obtained consistent results across priors. Based on coalescent theory and previous studies, priors specifying large ancestral *θ*s and recent divergences (*τ*) are expected to favor the recovery of fewer species in BP&P [25,43]. Moreover, if multiple prior combinations support one species delimitation while another prior scenario does not, then this may indicate that the data provide a poor fit to the latter prior, and *vice versa* e.g. [48]. Following Leaché & Fujita [43], we varied the prior distributions of population parameters estimated by BP&P by two orders of magnitude and found that all models unambiguously supported the same nine species.

Last, coalescent-based species delimitation approaches, like all species delimitation methods [5], are subject to the peculiarities of each study’s geographical, taxonomic, and character sampling strategies. Of particular concern are potentially negative effects of uneven sampling across distinct genetic lineages (e.g. the large bias toward sampling *P*. *mexicana* in clade 8 *versus* other clades) or unsampled taxa on coalescent-based species delimitations. Our sampling is the most comprehensive for the complex to-date at multiple levels, and we sampled most species recognized in the *P*. *sphenops* species complex prior to this study (S1 Table), except for four species with relatively restricted distributions, known from only 1 to 2 drainage basins in subregions of Mexico (*P*. *chica*, *P*. *marcellinoi*, *P*. *maylandi*) and Belize (*P*. *teresae*). We acknowledge our phylogenetic inferences are therefore subject to potential effects of missing species. In particular, without complete sampling, it is impossible to know whether lacking the above taxa has affected our guide tree topology in such a way that we have retained invalid nodes. However, lacking some ingroup taxa does not impact coalescent-based inferences of distinct species that are sampled e.g. [48], and given our BP&P analyses resulted in collapsing only a single node associated with phylogeographical structuring in clade 8 it seems unlikely that we have collapsed any valid nodes. Rather, undetected variation from unsampled species and populations might, at best, only influence inferred phylogeographical patterns and the positions of unsampled taxa within our gene trees and species trees.

### Hybridization *Versus* Incomplete Lineage Sorting

Identification of the species tree and species limits is a necessary prerequisite for understanding evolutionary genetic processes of hybridization-mediated introgression and incomplete lineage sorting, which are increasingly recognized in natural systems and thought to play a defining role influencing population genetic structure, speciation, and gene tree discordance [2,38,96]. Indeed, studies of these processes are vulnerable to the ‘species problem’, as they rely on defining species *a priori* before attempts are made to distinguish interspecific *versus* intraspecific processes [24]. Coalescent-based species delimitation provides a sound, objective basis for defining species for such analyses, which can provide important information reciprocally illuminating the nature of the species examined and conservation efforts [29]. Based on these methods, our study demonstrates a distinct pattern of nuclear, but not mitochondrial, hybridization and introgression, rather than ILS, as the main factor likely influencing gene tree discordance in the *P*. *sphenops* species complex. The presence of clear hybrid zones formed by post-speciation range expansion and secondary contact is a relatively common pattern in natural populations [2,5,19], but is not indicated in our results. Instead, we infer that some admixture has occurred in the past between species that today are sympatric and/or allopatric, as evidenced by smaller minimum pairwise nuclear genetic distances than that expected from posterior predictive distributions generated using coalescent simulations on species trees in JML [96]. Evidence seems especially complete for *P*. *butleri* hybridization (e.g. with *P*. *sphenops*/*catemaconis* in clade 2-a), as we detected introgression between *P*. *butleri* and other taxa at all nuclear loci analyzed (S1 Appendix). The available genetic evidence also apparently confirms previous morphological evidence for natural *P*. *butleri*–*P*. *sphenops* hybridization, including Schultz & Miller’s [50] description of a hybrid *P*. *butleri* × *P*. “*sphenops*” individual. Moreover, whereas *P*. *mexicana* has traditionally been considered to hybridize rarely with other *Poecilia* [50,51], our results support hybridization between this very widespread species (clade 8, Fig 1) and several other ingroup taxa (S1 Appendix).

Whereas maternally inherited mtDNA genomes are thought to generally introgress more rapidly and therefore to present poor bases for single-locus phylogenetics in various taxa including some fishes [103], our results overwhelmingly support cytonuclear discordance indicating the opposite is true for the *P*. *sphenops* species complex. This finding agrees with the expectation that nuclear gene flow and hybridization should be higher in systems with female-based dispersal, which is somewhat counterintuitive but supported by theory and empirical review by Petit & Excoffier [104]. Therefore, we hypothesize that a pattern of sexual asymmetry prevails in the *P*. *sphenops* species complex, with female-biased dispersal promoting intraspecific gene flow that blocks interspecific mtDNA introgression (*cf*. [104], references therein). This is the most plausible explanation for the patterns in our results, and underscores a contributing factor as to why mtDNA provide an excellent basis for species delimitation in the complex (as we have shown). In light of the above findings, that we observed consistency across our results and across species delimitation algorithms suggests that our species delimitations are robust to the effects of hybridization; we have also explicitly incorporated the effects of ILS during multiple modeling procedures, including species discovery and validation analyses.

### Phylogenetics and Biogeography

Though a more detailed comparison of our phylogenetic results and those of previous studies is beyond the scope of this study, we note that our findings agree with and expand on previous molecular hypotheses of phylogenetic relationships, hence inferred biogeography and diversification patterns, in the *P*. *sphenops* species complex [55,58,67,70]. For example, our multilocus phylogenies support both sub-complexes previously recognized within the complex *sensu lato* based on molecular and morphological studies [55,64] (S1 Table) as monophyletic (Figs 3 and 4 and S1B Fig). Still, morphological analyses are needed to determine whether the sub-complexes are reciprocally monophyletic, given we recover the candidate species *P*. sp. “Tipitapa” with morphological affinities for *P*. *mexicana* but undocumented dentition patterns as sister to the *P*. *sphenops* complex. Similar to Alda *et al*. [55], we found it difficult to obtain strongly supported relationships at some internodes of our species tree (e.g. relationships among clades 5–8), but results presented here and in [55] are congruent in suggesting that this has resulted from gene tree discordance caused by hybridization in the nuclear genome. By contrast, our six-gene dataset allowed us to obtain a species tree with better support for several relationships (with PP > 80–90) than [55]’s species tree. Moreover, we present the first multilocus species tree analysis strongly supporting the monophyly of the *P*. *sphenops* species complex and relationships within the *P*. *sphenops* complex (clades 1 and 2; Fig 4 and S1B Fig).

There are several major biogeographical implications of this study that go hand-in-hand with the taxonomic implications discussed below. First, our results clarify the geographical range limits of several taxa and thus aid combating the “Wallacean shortfall”, or gaps in our understanding of species distributions, in biodiversity studies [3]. Whereas others have considered *P*. *sphenops* to meet its southern range limit in eastern Guatemala or western Honduras [50,51,55,57], our results suggest that its range (i.e. of clade 2-b) extends further south, terminating at the lake district of Nicaragua, in Lake Nicaragua and its northern tributaries (Figs 1 and 4). Our results also clarify the distribution of ‘true’ *P*. *gillii* in clade 5 (the clade corresponding to the original type locality for this species, Río Chagres; see [55]), which it no longer makes sense to consider as spanning from Guatemala to Panama and perhaps into Colombia e.g. [59,66,99,102]. Instead, we recommend researchers and managers to consider the range of *P*. *gillii* as extending mainly from Río Playón Chico, Panama to Río Parismina, Costa Rica on the Atlantic versant, and from Río Bayano, Panama to around the western limit of the Río Térraba basin, Costa Rica on the Pacific versant (Figs 1 and 2). As in [55], we also find *P*. *mexicana* (clade 8) to have a much wider geographical distribution than previously thought e.g. [50,51]; however, given the uncertain status of Mexican populations in clade 7, we consider *P*. *mexicana* to extend from at least the Lake Petén Itzá drainage, Guatemala southward to Río Cuango, Panama on the Atlantic versant, and from Río Goascorán (the El Salvador-Guatemala border) to the western Río Bayano basin, Panama on the Pacific versant (Figs 1 and 2).

Second, the timing of diversification of the *P*. *sphenops* species complex inferred herein (Fig 4 and S1A Fig) is congruent with the results of previous fossil- and biogeography-calibrated, multilocus divergence time analyses by Alda *et al*. [55]. Particularly, our results based on expanded geographical and character sampling also show that lineage diversification has occurred *in situ* within Central America, and that all major-lineages diversified within the complex prior to the completion of the Isthmus of Panama, which connected North and South America ~3–1.8 Ma (reviewed in [105]). All nine delimited ‘species’ in our results fit this pattern (Fig 4), which is consistent with emplacement of the ancestral population of the complex through dispersal into the region from outlying areas of North or South America well before the full development of the Central American Isthmus landscape. We also inferred that the *P*. *mexicana* and *P*. *sphenops* complexes initially speciated during the Miocene (17.8–8.1 Ma; Fig 4 and S1 Fig), whereas multiple analyses with slightly different calibrations in [55] place the most recent common ancestor of these lineages in a slightly earlier Oligocene–Miocene range (~38–13 Ma), but overlap with our age estimates. These results correspond well to the results of [106], thus multiple datasets are apparently converging on a similar picture of the evolution of this group. Yet our discovery and coalescent-dating of the origin of the ‘cryptic’ species *P*. sp. “Tipitapa” from Nicaragua provides a unique insight: *in situ* evolution of this species ~9.2 Ma (Fig 4 and S1A Fig) correlates very closely with the origin of the Nicaraguan depression, which formed through southeast-northwestward opening of a rift valley between the Tortuguero lowlands of Costa Rica through the El Salvador Median Trough over 10–0 Ma (reviewed in [105]). This suggests that isolation in the Nicaraguan depression may have caused the initial divergence of this taxon.

Third, and more generally, we find evidence for both widespread and often-sympatric lineages (e.g. clades 2, 5, 8), as well as highly endemic lineages and phylogeographic units (e.g. clades 1, 2-a, 3, 4, and 5-a) (Figs 1 and 2). This suggests several contrasting biogeographical processes have been at play in shaping present-day distributions of species in the *P*. *sphenops* species complex. In particular, barriers between drainage basins (e.g. mountain ranges bounding the Nicaraguan depression) have apparently generated prolonged genetic isolation facilitating the development of distinct populations and endemic species within some regions, e.g. isolation of clade 1 within the Río San Juan basin. At the same time, dispersal barriers have been sufficiently negligible and time has been sufficiently great for some taxa, including *P*. *mexicana* and *P*. *gillii*, to obtain relatively extensive distributions across multiple biogeographical areas and physiographic provinces (reviewed in [105]), providing many opportunities for local adaptation and low levels of gene flow with sympatric congeners. These widespread lineages also inhabit very similar habitats [51,53,102], reflecting similar levels of phenotypic plasticity, and/or potentially large-scale ecological adaptation to similar environments.

### Taxonomic and Conservation Implications

Coalescent-based analyses such as those employed here should reduce investigator-driven biases in species delimitation, creating more stable and transparent taxonomy [29,43]. In making taxonomic interpretations based on our results, we follow a general lineage concept of species [10–12] and consider genealogical and statistical evidence from multiple unlinked genetic loci sufficient to diagnose independently evolving lineages representing distinct species [9,29,43]. This is considered best practice and is most consistent with recent progress in the conceptualization of species [12]. However, we acknowledge that evidence from species distributions indicating geographical isolation (e.g. allopatric ranges; [21]) and evidence for fixed morphological or ecological differences relative to other species can also support independent lineages as valid species (*cf*. [14–17,47]), though such differentiation is less likely to be observed in morphologically cryptic taxa.

Our coalescent-based species delimitation results support the distinctiveness of several existing *Poecilia* species. Most of the 9 lineages within the *P*. *sphenops* species complex delimited as strongly supported species correspond exclusively to nominal taxa and thereby support their continued recognition as distinct species. Specifically, we recognize *P*. *butleri*, *P*. *hondurensis*, and *P*. *mexicana* as distinct species, as presently defined, with the exception of considering *P*. *mexicana* to possess a more extensive range reaching Río Bayano, Panama (Figs 1 and 4; S1 Table). Coalescent species delimitation also non-subjectively delimits at least two undescribed candidate species within *P*. *mexicana*, including one new species in clade 1 (*P*. sp. “Tipitapa”), and two species within *P*. *gillii*, including the new species in clade 3 (“*gillii*” sp. 2), all of which are diagnosable based on molecular data including analyses of six independent loci. Fig 4 summarizes the placement of each of these lineages in the species tree, and Fig 1 provides a map of each lineage’s distribution in a regional context. Our interpretation that at least two species exist within *P*. *gillii* is conservative, given the species we consider ‘true’ *P*. *gillii* in clade 5 contains three sub-lineages, each of which was strongly supported in phylogenetic analyses of the mtDNA and concatenated mtDNA + nDNA datasets, though not delimited during GMYC species discovery analyses. Although *P*. *mexicana* and *P*. *gillii* vary substantially in pigmentation and dorsal fin coloration throughout their ranges [59,102], we are aware of very few morphological characters or ecological attributes distinguishing the two new candidate species within *P*. *mexicana*, and aware of no such attributes distinguishing the two species within *P*. *gillii*. However, as these species are already strongly supported by multilocus molecular data, studies exploring their distributions, ecological niches, and morphology in further detail would provide additional support for their validity (*cf*. [43]). Thus, we recommend that a formal morphological description of each candidate species be undertaken, including an analysis of all related type material and morphological comparisons with closely related species.

## Conclusions

Overall, our findings contribute to a growing appreciation of the utility of combining multiple lines of genetic evidence and broad phylogeographical sampling to discover and validate species limits using coalescent-based methods [29,43,93]. Our study also contributes to a more accurate accounting of the biodiversity and geographical distributions of *Poecilia* mollies (subgenus *Mollienesia*), as well as Central American freshwater fishes in general, through objectively delimiting species in the *P*. *sphenops* species complex using molecular data. The importance of testing for hybridization *versus* ILS on multilocus species trees is also highlighted by our results: distinguishing between these factors allowed us to infer not only species boundaries but also evolutionary processes influencing genetic diversity in the complex, as well as our inferences. In particular, our data support the hypothesis that cytonuclear discordance arises in this complex as a result of female-biased dispersal (although we cannot rule out at least some mtDNA introgression). We recommend additional sampling of *P*. *sphenops* species complex populations at additional unlinked genetic loci to further improve the taxonomy and biogeography of the group and achieve a phylogenetic analysis with more complete ingroup sampling; however, we highlight the importance of our findings to understanding the biogeographical processes influencing this group, as well as their significance for taxonomy and conservation.

## Supporting Information

S1 Appendix Supplementary methods and results. (DOCX)

S1 Data Taxon list and locality (sub-population) details. (XLSX)

S1 Fig Map showing geographical coverage of mtDNA subsamples in the concatenated mtDNA dataset. (EPS)

S2 Fig Map showing geographical coverage of nDNA subsamples in the concatenated nDNA dataset. (EPS)

S3 Fig BEAST MCC tree derived from the concatenated mtDNA dataset. (EPS)

S4 Fig Gene trees of nuclear DNA loci sampled for gene tree, species, tree, and species delimitation analyses. Each delimited species is coded the corresponding clade color from Fig 2. Scale bars are in units of substitutions per site. (EPS)
(EPS)

S5 Fig Matching tree and posterior probability matrix from Bayesian general mixed Yule-coalescent bGMYC analyses. The phylogeny is the BEAST MCC tree (S1 Fig) and the tables at right of each tree provide sequence-by-sequence visualizations of the posterior probability that each species/sequence pair is conspecific. (A) Results from the single-threshold model. (B) Results from the multiple-threshold model. (EPS)
(EPS)

S6 Fig Species tree showing posterior probabilities of species for each node under each combination of priors using algorithm 0 in BP&P. The red line and nodal values correspond to the same criteria and priors described in Fig 4. (EPS)
(EPS)

S1 Table Summary of the taxonomy, tooth morphology, and distributions of species in the *Poecilia sphenops* species complex. (DOCX)

S2 Table DNA substitution models selected using DT-ModSel. (DOCX)

S3 Table Sequence attributes and DNA polymorphism levels in each of the datasets analyzed in this study, overall and by gene. (DOCX)
