# Supplementary material for: Assessing Species Boundaries Using Multilocus Species Delimitation in a Morphologically Conserved Group of Neotropical Freshwater Fishes, the Poecilia sphenops Species Complex (Poeciliidae)
Source: PLoS One. 2015 Apr 7;10(4):e0121139. doi: 10.1371/journal.pone.0121139 (PMC4388586; doi:10.1371/journal.pone.0121139)
Supplement: S1 Appendix — (DOCX) [file pone.0121139.s001.docx]

**Appendix S1** Supplementary methods and results.

**Taxon sampling and sequencing, and outgroup details**

Here, we provide additional sequence data and outgroup descriptions relevant to our analyses but not listed in the main text. As noted in the manuscript, our individual analyses utilized up to 21 tips representing outgroup taxa, and the sample data for each outgroup are given in table format in supplementary Data S1. However, the pool of outgroup taxa spanned 23 ‘outgroup’ tips representing 15 nominal poeciliid taxa, including (1) *Poecilia caucana*, the sister taxon to the members of the *P. sphenops* species complex (based on analyses by Alda *et al.* [1]); the sail-fin mollies (2) *P. latipinna* and (3) *P. latipunctata*; the South American guppies (4) *Micropoecilia picta* and (5) *Poecilia reticulata*; the Mexican swordtails (6) *Xiphophorus helleri* and (7) *X. maculatus*; the Central American Pike Killifish, (8) *Belonesox belizanus*; and six species of fishes from the genus *Limia*, a closely related genus whose members were formerly included within *Poecilia* subgenus *Limia*: (9) *L. dominicensis*, (10) *L. melanogaster*, (11) *L. melanonotata*, (12) *L. tridens*, (13) *L. vittata*, (14) *L. heterandria*, and (15) *L. perugiae*. GenBank numbers for the sequences we used to represent these outgroup taxa are provided in Data S1. A total of 21 outgroup samples representing the first 13 of these outgroup taxa were used in phylogenetic analyses of our ‘concatenated mtDNA’ dataset, including the BEAST relaxed clock analysis whose results are presented in supplementary Fig. S3A. However, our ‘concatenated nDNA’ dataset included only 7 outgroup tips representing the following five species: *P. latipinna*, *P. latipunctata*, *M. picta*, *P. reticulata*, and *L. perugiae*; thus, concatenated gene trees from analyses of this dataset included up to five outgroup species, though only *Poecilia* are shown in the resulting figures (e.g. Fig. 3). As mentioned in the main text, our *BEAST analyses of the ‘concatenated mtDNA + nDNA’ dataset included outgroup samples from 15 species. These 15 outgroup lineages consisted of all 15 of the outgroup species listed above; again, most of these outgroups except selected *Poecilia* were pruned from the trees resulting from the analyses before finalizing our figures (e.g. Fig. S3B).

Regarding PHASE analyses, *Glyt* and X-*yes* alignments could not be completely resolved due to multiple positions with >2 variants per position, so we analyzed phased alleles for all other loci and coded ambiguities in the *Glyt* and X-*yes* alignments as missing. Iterative analyses (e.g. using all six loci in BEAST) using alignments for which we had arbitrarily resolved ambiguities in the data for these two loci did not give results that were significantly different than those presented in the manuscript (data not shown).

**Neutrality and recombination**

Consistent with expectations of neutral evolution, which was assumed in all of our analyses, Hudson-Kreitman-Aguadé tests [2] were non-significant for the full-cyt*b* dataset (N = 938 ingroup sequences, χ^2^ = 0.027, *P* = 0.87), the cyt*b* matrix from the concatenated mtDNA dataset (N = 134 ingroup sequences, χ^2^ = 0.046, *P* = 0.83), and the *cox1* matrix from the concatenated mtDNA dataset (N = 111 ingroup sequences, χ^2^ = 0.058, *P* = 0.81). One *P. caucana* outgroup sample was used in each HKA test.

For additional insight into neutrality and in an attempt to cross-validate the HKA test results, we also ran McDonald & Kreitman tests [3] in DnaSP on the same mtDNA datasets. One *P. caucana* outgroup sample was used in each MK test. Similar to HKA tests, the MK tests also supported neutrality of the mtDNA data in analyses of the full-cyt*b* dataset (N = 938 ingroup sequences, alpha = 0.11, *P*_Fisher_ = 0.818; *G* = 0.067, *P* = 0.796) and the *cox1* matrix from the concatenated mtDNA dataset (N = 111 ingroup sequences, alpha = 1.00, *P*_Fisher_ = 1.00; *G*-test could not be performed). However, an MK test on the cyt*b* matrix from the concatenated mtDNA dataset was significant (N = 134 ingroup sequences, alpha = 0.723, *P*_Fisher_ = 0.0026; *G* = 8.823, *P* = 0.0030). To further evaluate whether the non-neutral signal in the concatenated mtDNA cyt*b* matrix may have resulted from past population dynamics, e.g. population genetic bottlenecking or expansion, rather than selection we conducted additional coalescent simulations on this dataset in DnaSP (again, testing significance with 1000 simulations) using the neutrality statistics Fu’s *F*_S_ and *R*_2_ [4]. We estimated a negative value of *F*_S_ for this dataset indicating potential past population growth, but this result was non-significant (mean *F*_S_ = −1.102, 95% confidence interval = [−14.855, 11.437], *P* = 0.593). However, a positive and significant *R*_2_ value (mean *R*_2_ = 0.088, 95% confidence intervals = [0.047, 0.145], *P* < 0.001) indicated that the non-neutral signal in the concatenated mtDNA cyt*b* dataset may owe to past population expansion rather than selection. In view of these results, further testing using analogous coalescent simulations of Fay & Wu’s [5] *H* in DnaSP was used to evaluate whether the hypothesis of positive selection could be ruled out. The results were consistent with the interpretation that the MK and neutrality test results reported above for the concatenated mtDNA cyt*b* dataset were not influenced by positive selection, e.g. due to hitchhiking (mean *H* = −1.350, 95% confidence interval = [−77.191, 28.226], *P* = 0.350). Overall, the various mtDNA analyses above indicate that all of the mtDNA data used in this study are selectively neutral, though likely influenced by historical demographic fluctuations. As a result, it seems worthwhile to delve further into this issue with analyses targeted at better understanding the historical demography of the *P. sphenops* species complex.

We ran seven different tests for recombination on each of the nuclear loci analyzed in this study. Six tests were run on each of the loci simultaneously in the program RDP3 (citation in main text; default parameters unless stated otherwise, in parentheses below) using different algorithms for recombination detection, including the original RDP method ([6]; window size = 30), the GENCONV Local method [7], the RecScan/Bootscan method ([8]; window size = 100, step size = 20,500 bootstrap replicates), the MaxChi Local method [9], the Chimaera method [10], and the 3seq method [11]. Among all 30 tests run RDP3 (six algorithms run on each of 5 loci), we recovered evidence for only three unique events corresponding to three recombination signals, which were only discovered by three methods (MaxChi, Chimaera, 3seq) when analyzing the X-*yes* dataset. All other tests conducted in RDP3 for X-*yes* and the other loci inferred zero events/signals.

Runs of our seventh test for recombination using coalescent simulations in DnaSP assumed intermediate levels of recombination (*R*, per gene) and empirical mutation parameter θ (per gene). The simulations were run based on an implementation of the coalescent based on Hudson [12], and DnaSP obtained the estimated *R*-values using the method of Hudson [13], whereas observed RM estimates were obtained using equations in Hudson & Kaplan [14]. Estimated values of *R* used in the simulations were, by gene, as follows: *ldh-A*, 0.499; *RPS7*, 0.399; X-*src*, 4.099; X-*yes*, 0.001; *Glyt*, 47.299. Empirically estimated minimum numbers of recombination events (*R*_M_) calculated directly from the data were, by gene, as follows: *ldh-A*, 0; *RPS7*, 5; X-*src*, 9; X-*yes*, 10; *Glyt*, 5. The results of these coalescent simulations were non-significant, indicating less recombination than expected. Specifically, the probabilities of recombination being less than or equal to the minimum number of events (*P*-values) were each non-significant at the test level (α = 0.05): *ldh-A*, *P* = 0.87; *RPS7*, *P* = 1.00; X-*src*, *P* = 1.00; X-*yes*, *P* = 1.00; *Glyt*, *P* = 0.58.

**Coalescent-based species delimitation**

As noted in the Discussion section, we conducted additional analyses to evaluate the potential effects of phylogenetic branch lengths and their uncertainty on our GMYC-based species discovery analysis. Specifically, we tested whether an algorithm similar to bGMYC, but not relying heavily on phylogenetic branch lengths in units of time, gave comparable results to our preliminary species delimitation hypotheses shown in Fig. 2. To accomplish this, we delimited species on our data using a Bayesian implementation of PTP [15], which uses a “Poisson tree process” to model speciation rates and detect the transition point between within-species branching processes and between-species branching processes. PTP was suitable for our purposes because it analyzes substitution patterns along gene tree branches without requiring an ultrametric topology, and without utilizing branch length information in units of time, which may be prone to potential error or biases [15]. Thus, PTP avoids confounding effects of time-calibrated branch length uncertainty [15]. The PTP algorithm also happens to be fast and intuitive to implement. Bayesian PTP analyses were run on the concatenated mtDNA ML gene tree from GARLI shown in Fig. 2, which, importantly, is similar to the MCC tree that we analyzed in all of our bGMYC runs (Fig. S3). We ran our PTP analysis using the “bPTP.py” python script implemented on the PTP web server (<http://species.h-its.org/ptp/>). As noted in the text, we found that Bayesian PTP gave species delimitations that were nearly identical to our bGMYC-delimited species (data not shown). Thus, we conclude that our mtDNA data are robust to species delimitation using methods with and without taking branch lengths into account. However, it remains unclear whether and to what extent uneven sampling across distinct species lineages may have influenced these species discovery analyses. Although evaluating such properties of the data and the bPTP algorithm are beyond the scope of the present study, we expect that these topics will be addressed using simulations in future studies.

**Evolutionary rates estimated in *BEAST, used in JML analyses**

The main *BEAST [16] analysis in BEAST v2.0.2 [17] employed relaxed clocks for all loci and two fossil/biogeographical calibration points described in the main text. Based on five independent runs conducted during this analysis, we inferred the following evolutionary substitution rates for each locus: concatenated mtDNA, 0.005656; *ldh-A*, 0.001768; *RPS7*, 0.0009142; X-*src*, 0.0004724; X-*yes*, 0.005094; *Glyt*, 0.0002984. Each of these rates is a mean estimate in units of substitutions per site per million years (subs/site/myr), per lineage. Note the mtDNA rate fell within the uniform ‘fish rate’ prior set on the locus for this analysis (0.0017–0.014 subs/site/myr), as expected.

We ran a second *BEAST analysis, again based on five independent runs, specifying independent relaxed clocks for each locus, but no calibration points. This analysis was conducted in order to facilitate simulation analyses in JML [18], and calibration points were not available because only ingroup taxa in the *P. sphenops* species complex were included in the analysis, as warranted by our JML analyses. From this second *BEAST analysis, we inferred the following mean relative rate estimates for each locus: concatenated mtDNA, 1.049; *ldh-A*, 0.652; *RPS7*, 0.282; X-*src*, 0.166; X-*yes*, 0.473; *Glyt*, 0.08112. In contrast to the rates reported for our main *BEAST analysis above, these values are relative evolutionary rates for each gene partition (which were unlinked), estimated relative to the mean rate. Values from this set of relative rates were supplied to JML during coalescent simulations used to test for hybridization versus incomplete lineage sorting in the *ldh-A*, *RPS7*, and X-*src* loci.

**Full/additional JML results**

In the main text, we highlight the most consistently recovered patterns among our JML results. Here, we give JML results for each of the three nDNA loci analyzed using posterior predictive testing analyses. We list species pairs for which we detected evidence of introgression, as well as ranges or point values of significant *P*-values for the test statistic, *minDist* [18]. JML simulations detected introgressed nuclear *ldh-A* sequences between *P. gillii-P. hondurensis*, *P. butleri*-*P. catemaconis/sphenops* (clade 2-a), *P. mexicana*-*P. catemaconis/sphenops*, *P. mexicana-P. hondurensis*, and *P. mexicana*-*P. butleri* species pairs (*minDist* *P* = 0.001–0.048). JML simulations also detected introgressed *RPS7* sequences between *P. hondurensis-P.* sp. “Tipitapa”, *P. hondurensis*-*P. catemaconis/sphenops*, *P. hondurensis-P. sphenops* (clade 2-b), *P. butleri*-*P. catemaconis/sphenops*, *P. mexicana-P. catemaconis/sphenops*, and *P. mexicana-P. butleri* species pairs (*minDist* *P* = 0.001–0.032). Last, introgressed X-*src* sequences were detected between the *P. butleri*-*P. catemaconis/sphenops* species pairs (*minDist* *P* = 0.001). Results from clade 7 are not included in the above lists, because a lack of observed sequence data for nuclear loci prohibited us from calculating exact *minDist* probabilities for this clade.

**References**

1. Alda FA, Reina RG, Doadrio I, Bermingham E (2013) Phylogeny and biogeography of the *Poecilia sphenops* species complex (Actinopterygii, Poeciliidae) in Central America. Molecular Phylogenetics and Evolution 66:1011-1026.
2. Hudson RR, Kreitman M, Aguadé M (1987) A test of neutral molecular evolution based on nucleotide data. Genetics 116:153-159.
3. McDonald JH, Kreitman M (1991) Adaptive protein evolution at the *Adh* locus in *Drosophila*. Nature 351:652-654.
4. Ramos-Onsins SE, Rozas J (2002) Statistical properties of new neutrality tests against population growth. Molecular Biology and Evolution 19:2092-2100.
5. Fay JC, Wu CI (2000) Hitchhiking under positive Darwinian selection. Genetics 155:1405-1413.
6. Martin D, Rybicki E (2000) RDP: detection of recombination amongst aligned sequences. Bioinformatics 16:562-563.
7. Padidam M, Sawyer S, Fauquet CM (1999). Possible emergence of new geminiviruses by frequent recombination. Virology 265:218-225.
8. Martin DP, Posada D, Crandall KA, Williamson C (2005) A modified BOOTSCAN algorithm for automated identification of recombinant sequences and recombination breakpoints. AIDS Research and Human Retroviruses 21:98-102.
9. Maynard Smith J (1992). Analyzing the mosaic structure of genes. Journal of Molecular Evolution 34:126-129.
10. Posada D, Crandall KA (2001). Evaluation of methods for detecting recombination from DNA sequences: Computer simulations. Proceedings of the National Academy of Sciences of the United States of America 98:13757-13762.
11. Boni MF, Posada D, Feldman MW (2007). An exact nonparametric method for inferring mosaic structure in sequence triplets. Genetics 176:1035-1047.
12. Hudson RR (1990) Gene genealogies and the coalescent process. Oxford Surveys in Evolutionary Biology 7:1-44.
13. Hudson RR (1987) Estimating the recombination parameter of a finite population model without selection. Genetic Research 50:245-250.
14. Hudson RR, Kaplan NL (1985) Statistical properties of the number of recombination events in the history of a sample of DNA sequences. Genetics 111:147-164.
15. Zhang J, Kapli P, Pavlidis P, Stamatakis A (2013) A general species delimitation method with applications to phylogenetic placements. Bioinformatics 29:2869-2876.
16. Heled J, Drummond AJ (2010) Bayesian inference of species trees from multilocus data. Molecular Biology and Evolution 27:570-580.
17. Bouckaert R, Heled J, Kühnert D, Vaughan TG, Wu C-H, Xie D, Suchard MA, Rambaut A, Drummond AJ (2014) BEAST2: A software platform for Bayesian evolutionary analysis. PLoS Computational Biology. Available at: http://beast2.org/.
18. Joly S (2012) JML: testing hybridization from species trees. Molecular Ecology Resources 12:179-184.
