# Supplementary material for: Assessing Species Boundaries Using Multilocus Species Delimitation in a Morphologically Conserved Group of Neotropical Freshwater Fishes, the Poecilia sphenops Species Complex (Poeciliidae)
Source: PLoS One. 2015 Apr 7;10(4):e0121139. doi: 10.1371/journal.pone.0121139 (PMC4388586; doi:10.1371/journal.pone.0121139)
Supplement: S1 Table — (DOCX) [file pone.0121139.s009.docx]

**Table S1** Summary of the taxonomy, tooth morphology, and distributions of species in the *Poecilia sphenops* species complex.

|  |  | **Taxonomic hypotheses** | | |  |  | **Biogeography** | |
| --- | --- | --- | --- | --- | --- | --- | --- | --- |
| **Taxon name** | **Common name** | **^§^Rosen & Bailey [1]** | **^†^Schultz & Miller [2]; Miller [3]** | **^¶^Alpírez Quesada [4]** | **Current status ([3,5,6,9])** | **Inner jaw teeth** | **Versant** | **Distribution [2–9]** |
| **Described species and subspecies** | |  |  |  |  |  |  |  |
| ***Poecilia butleri* Jordan 1889***** | Pacific Molly | ­*P. sphenops* | ­*P. sphenops* species complex | *P. mexicana* complex | Valid | uni. | Pacific | Mexico to El Salvador |
| ***P. catemaconis* Miller 1975***** | Catemaco Molly | ­*P. sphenops* | ­*P. sphenops* species complex | *P. mexicana* complex | Valid | uni. | Atlantic | Lake Catemaco, Mexico |
| ***P. chica* Miller 1975** | Dwarf Molly | ­*P. sphenops* | ­*P. sphenops* species complex | *P. sphenops* complex | Valid | tri. | Pacific | Basins of Cuetzamala River and Purificación in Jalisco, Mexico |
| ***P. gillii* (Kner 1863)***** | Gill’s Molly | ­*P. sphenops* | ­*P. sphenops* species complex | *P. mexicana* complex | Valid | uni. | Atlantic, Pacific | Atlantic versant from Guatemala to Colombia, along the Pacific versant from Guatemala to the Terrába River, Costa Rica, and from the Grande River to the Bayano River in Panama |
| ***P. hondurensis* Poeser 2011***** | Honduras Molly | ­*P. sphenops* | ­*P. sphenops* species complex | *P. mexicana* complex | Valid | uni. | Atlantic | Caribbean drainages of Honduras |
| ***P. marcellinoi* Poeser 1995** | Molly | ­*P. sphenops* | ­*P. sphenops* species complex | *P. sphenops* complex | Valid | tri. | Pacific | Ilopango Lake basin, El Salvador |
| ***P. maylandi* Meyer 1983** | Balsas Molly | ­*P. sphenops* | ­*P. sphenops* species complex | *P. sphenops* complex | Valid | tri. | Pacific | Balsas River basin and Aguililla River, Mexico |
| ***P. mexicana* Steindachner 1863***** | Shortfin Molly | ­*P. sphenops* | ­*P. sphenops* species complex | *P. mexicana* complex | Valid | uni. | Atlantic, Pacific | Atlantic versant from northeastern Mexico to Costa Rica and in the Río Tamarindo, in the Pacific slope of Nicaragua |
| - ***P. m. mexicana* Steindachner 1863***** | Shortfin Molly | ­*P. sphenops* | ­*P. sphenops* species complex | *P. mexicana* complex | Synonym of *P. mexicana*; however, Menzel & Darnell [5] have recommended subspecies rank and suggested it intergrades with another subspecies (*P. m. limantouri*) in eastern Mexico | uni. | Atlantic, Pacific | Río Cazones south (at least) to Río Jamapa system in eastern Mexico |
| - ***P. m. limantouri* Jordan & Synder 1901***** | Limantour’s Molly | ­*P. sphenops* | ­*P. sphenops* species complex | *P. mexicana* complex | Synonym of *P. mexicana* [1]; however, others have recommended subspecies rank and suggested it intergrades with *P. m. mexicana* in eastern Mexico [5] | uni. | Atlantic | Southern Río Grande and Río San Fernando headwaters, south to Pánuco River, Mexico |
| ***P. orri* Fowler 1943***** | Mangrove Molly | ­*P. sphenops* | ­*P. sphenops* species complex | *P. mexicana* complex | Valid | uni. | Atlantic | Western coasts of Yucatan Peninsula southeast to northern Honduras |
| ***P. salvatoris* Regan 1907** | Salvador Molly | ­*P. sphenops* | ­*P. sphenops* species complex | *P. mexicana* complex | Valid | uni. | Pacific | El Salvador |
| ***P. sphenops* Valenciennes 1846***** | Mexican Molly | ­*P. sphenops* | ­*P. sphenops* species complex | *P. sphenops* complex | Valid | tri. | Atlantic, Pacific | Atlantic slope of Mexico from the Palma Sola River to the Grijalva River basin, and along the Pacific slope from the Río Verde basin into Guatemala |
| ***P. sulphuraria* (Álvarez 1948)***** | Sulphur Molly | ­*P. sphenops* | ­*P. sphenops* species complex | *P. mexicana* complex | Valid | uni. | Atlantic | Baños del Azufre, near Teapa, Tabasco, Mexico |
| ***P. teresae* Greenfield 1990** | Mountain Molly | ­*P. sphenops* | ­*P. sphenops* species complex | *P. mexicana* complex | Valid | uni. | Atlantic | Mountain Pine Ridge, Mayan Mountain Range, Belize |
|  |  |  |  |  |  |  |  |  |
| **Molecular operational taxonomic units (OTUs)** | | |  |  |  |  |  |  |
| **“*sphenops*” sp. 1*** | – | – | – | *–* | Candidate species ([6]; this study) | – | Atlantic, Pacific | Río Goascorán and Río Ulúa, Honduras |
| **“*gillii*” sp. 2*** | – | – | – | *–* | Candidate species ([6]; this study) | – | Atlantic | Río Acla, Panama |
| ***P*. sp. “Patuca”** | – | – | – | *–* | Considered part of the “*P. gillii*” lineage, clade 5 (this study) | – | Atlantic | Río Patuca basin, Honduras |
| ***P*. sp. “Tipitapa”** | – | – | – | *–* | Candidate species (this study) | – | Atlantic | Río Tipitapa and northern Lake Nicaragua tributaries, Río San Juan basin, Nicaragua |

Asterisks placed by taxon names indicate nominal taxa or molecular OTUs previously recognized in the *P. sphenops* species complex by other authors, and that we also sampled in our study. This table also presents data from [6] on differences in inner jaw tooth morphology displayed among taxa from the species complex (uni., unicuspid; tri., tricuspid).

^§^Interpretation recognizing the existence of a single, polytypic species; this study synonymized various taxa under *P. sphenops*.

^†^Interpretation recognizing multiple species forming a single “*P. sphenops* species complex.”

^¶^Interpretation recognizing two species groups or complexes, the “*P. sphenops* complex” and the “*P. mexicana* complex”, within the *P. sphenops* species complex *sensu lato*.

**References**

1. Rosen DE, Bailey RM (1963) The poeciliid fishes (Cyprinodontiformes), their structure, zoogeography, and systematics. Bulletin of the American Museum of Natural History 126:1-176.
2. Schultz RJ, Miller RR (1971) Species of the *Poecilia sphenops* complex (Pisces: Poeciliidae) in Mexico. Copeia 1971:282-290.
3. Miller RR (2005) *Freshwater Fishes of México*. The University of Chicago Press, Chicago.
4. Alpírez Quesada O (1971) Estudio sistemático del complejo *Poecilia sphenops* (Familia Poeciliidae) de Centroamérica en especial de las poblaciones de Costa Rica. Universidad de Costa Rica, San José.
5. Menzel BW, Darnell RM (1973) Systematics of *Poecilia mexicana* (Pisces: Poeciliidae) in Northern Mexico. Copeia 1973:225-237.
6. Alda FA, Reina RG, Doadrio I, Bermingham E (2013) Phylogeny and biogeography of the *Poecilia sphenops* species complex (Actinopterygii, Poeciliidae) in Central America. Molecular Phylogenetics and Evolution 66:1011-1026.
7. Matamoros WA, Kreiser BR, Schaefer JF (2012) A delineation of Nuclear Central America biogeographical provinces based on river basin faunistic similarities. Reviews in Fish Biology and Fisheries 22:351-365.
8. Bussing WA (1998) *Freshwater Fishes of Costa Rica*, 2nd Edn. Editorial de la Universidad de Costa Rica, San José, Costa Rica.
9. Poeser FN (2011) A new species of *Poecilia* from Honduras (Teleostei: Poeciliidae). Copeia 2011:418-422.
