# Supplementary material for: Assessing Species Boundaries Using Multilocus Species Delimitation in a Morphologically Conserved Group of Neotropical Freshwater Fishes, the Poecilia sphenops Species Complex (Poeciliidae)
Source: PLoS One. 2015 Apr 7;10(4):e0121139. doi: 10.1371/journal.pone.0121139 (PMC4388586; doi:10.1371/journal.pone.0121139)
Supplement: S2 Table — (DOCX) [file pone.0121139.s010.docx]

**Table S2 DNA substitution models selected using DT-ModSel.**

| DNA dataset | Partition | *n* | bp | Best model | Analysis |
| --- | --- | --- | --- | --- | --- |
| **Full-cyt*b*** | |  |  |  |  |
|  | All Cyt*b* (analyzed together, or as *n* = 260 haplotypes) | 941 | 1086 | TVM+*Γ+I* | tcs, DnaSP |
| **Concatenated mtDNA** | |  |  |  |  |
|  | 1^st^ + 2^nd^ codon positions | 171 | 590 | HKY+*Γ+I* | garli, MrBayes, beast, DnaSP |
|  | 3^rd^ codon position | 171 | 1180 | GTR+*Γ* | garli, MrBayes, beast, DnaSP |
| **Concatenated nDNA (overall *n* = 50)** | | | | | |
|  | *ldh-A* | 42 | 191 | JC+*I* | garli, bpp |
|  | *RPS7* | 44 | 1158 | K80+*Γ*+*I* | garli, bpp |
|  | X-*src* | 45 | 518 | K80+*I* | garli, bpp |
|  | X-*yes* | 20 | 833 | HKY+*I* | garli, bpp |
|  | *Glyt* | 21 | 915 | K80+*I* | garli, bpp |
| **Concatenated mtDNA + nDNA (overall *n* = 80)** | |  |  |  |  |
|  | mtDNA 1^st^ + 2^nd^ codon positions | 80 | 590 | TVM+*Γ*+*I* | garli, MrBayes, beast (*BEAST) |
|  | mtDNA 3^rd^ codon position | 80 | 1180 | TrN+*Γ*+*I* | garli, MrBayes, beast (*BEAST) |
|  | mtDNA 1^st^ + 2^nd^ codon positions, reduced dataset* | 50 | 590 | HKY+*Γ* | beast (*BEAST) |
|  | mtDNA 3^rd^ codon position, reduced dataset* | 50 | 1180 | TrN+*Γ* | beast (*BEAST) |
|  | *ldh-A* | 42 | 191 | JC+*Γ*+*I* | garli, MrBayes, beast (*BEAST) |
|  | *RPS7* | 44 | 967 | HKY+*Γ* | garli, MrBayes, beast (*BEAST) |
|  | X-*src* | 45 | 518 | K80+*I* | garli, MrBayes, beast (*BEAST) |
|  | X-*yes* | 20 | 833 | HKY+*Γ* | garli, MrBayes, beast (*BEAST) |
|  | *Glyt* | 21 | 915 | K80+*I* | garli, MrBayes, beast (*BEAST) |

Model selection analyses using the decision theory algorithm in DT-ModSel [1] supported different best-fit models of DNA evolution for different datasets, including datasets filtered by codon partitions. We preferred DT-ModSel for our substitution model selection analyses, rather than other model selection software, because DT-ModSel has been shown to recover models that yield superior ML branch lengths relative to other comparable programs [1]. This table lists model selection results for each dataset analyzed in this study, as well as the analyses that each dataset (thus molecular model, wherever possible) was used in. Symbols and abbreviations: *Γ*, gamma-distributed rate variation; bp, number of nucleotide base pairs; DnaSP, DNA polymorphism and neutrality statistics analyses conducted in the program by the same name; *I*, parameter representing proportion of invariable sites; ML, maximum likelihood phylogenetic analyses estimating gene trees; *n*, sample sizes for each locus or gene. Overall alignment sizes are either the maximum *n* reported for each dataset, or given in parentheses next to the dataset name. Asterisks in the “Partition” column denote mitochondrial datasets that are reduced versions of the 86-taxon mtDNA dataset, and which we used (along with the nDNA in the concatenated mtDNA + nDNA dataset) in the *BEAST analyses ran to create a posterior distribution of species trees with appropriate tips for analysis in JML.

**References**

1. Minin V, Abdo Z, Joyce P, Sullivan J (2003) Performance-based selection of likelihood models for phylogeny estimation. Syst Biol 52: 674-683.
