# Supplementary material for: Assessing Species Boundaries Using Multilocus Species Delimitation in a Morphologically Conserved Group of Neotropical Freshwater Fishes, the Poecilia sphenops Species Complex (Poeciliidae)
Source: PLoS One. 2015 Apr 7;10(4):e0121139. doi: 10.1371/journal.pone.0121139 (PMC4388586; doi:10.1371/journal.pone.0121139)
Supplement: S3 Table — (DOCX) [file pone.0121139.s011.docx]

**Table S3 Sequence attributes and DNA polymorphism levels in each of the datasets analyzed in this study, overall and by gene.**

| DNA dataset | Partition | *n* | bp | Variable characters (%) | Parsimony informative characters (%) | Overall mean *d* (s.e.) |
| --- | --- | --- | --- | --- | --- | --- |
| **Full-cyt*b*** | |  |  |  |  |  |
|  | All cyt*b* ingroup sequences | 941 | 1086 | 341 (31.40) | 301 (27.72) | 0.026 (0.0020) |
| **Concatenated mtDNA** | | 171 |  |  |  |  |
|  | 1^st^ + 2^nd^ codon positions, ingroup only | 147 | 1180 | 213 (18.05) | 131 (11.10) | N/A |
|  | 3^rd^ codon position, ingroup only | 147 | 590 | 233 (39.49) | 209 (35.42) | N/A |
|  | All cyt*b* | 155 | 1086 | 460 (42.36) | 403 (37.11) | 0.064 (0.0031) |
|  | All *cox1* | 115 | 684 | 206 (30.12) | 128 (18.71) | 0.032 (0.0030) |
| **Concatenated nDNA** | |  |  |  |  |  |
|  | *ldh-A* | 42 | 191 | 19 (9.94) | 14 (7.32) | 0.025 (0.0098) |
|  | *RPS7* | 44 | 1158 | 141 (12.18) | 126 (10.88) | 0.018 (0.0020) |
|  | X-*src* | 45 | 518 | 33 (6.37) | 25 (4.83) | 0.0095 (0.0022) |
|  | X-*yes* | 20 | 833 | 89 (10.68) | 53 (6.36) | 0.025 (0.0030) |
|  | *Glyt* | 21 | 915 | 30 (3.28) | 14 (1.53) | 0.0056 (0.0011) |
| **Concatenated mtDNA + nDNA*** | |  |  |  |  |  |
|  | Concatenated mtDNA | 80 | 1770 | 661 (37.34) | 660 (37.29) | N/A |
|  | mtDNA 1^st^ + 2^nd^ codon positions | 80 | 1180 | 334 (28.31) | 333 (28.22) | N/A |
|  | mtDNA 3^rd^ codon position | 80 | 590 | 327 (55.42) | 327 (55.42) | N/A |

This table presents results from DNA sequence analyses in mega5 [1]. ‘Overall mean *d*’ values are estimates of the average evolutionary distance over all sequence pairs, calculated using *p*-distances (base differences per site), and are given with their standard errors (s.e.) shown in parentheses based on 500 bootstrap pseudoreplicates. Sites with <95% site coverage were eliminated prior to *d* calculations. *n*, sample size; N/A, not available.

*The concatenated nDNA dataset was also analyzed along with these mtDNA in analyses of the ‘concatenated mtDNA + nDNA’ dataset, and their results are not duplicated here because they are the same as above (under “Concatenated nDNA”).

**References**

1. Tamura K, Peterson D, Peterson N, Stecher G, Nei M, Kumar S (2011) MEGA5: molecular evolutionary genetics analysis using maximum likelihood, evolutionary distance, and maximum parsimony methods. Molecular Biology and Evolution 28:2731-2739.
